# Differential Phosphorylation of Ribosomal Proteins in *Arabidopsis thaliana* Plants during Day and Night

**DOI:** 10.1371/journal.pone.0029307

**Published:** 2011-12-16

**Authors:** Maria V. Turkina, Hanna Klang Årstrand, Alexander V. Vener

**Affiliations:** Division of Cell Biology, Department of Clinical and Experimental Medicine, Linköping University, Linköping, Sweden; Instituto de Biología Molecular y Celular de Plantas, Spain

## Abstract

Protein synthesis in plants is characterized by increase in the translation rates for numerous proteins and central metabolic enzymes during the day phase of the photoperiod. The detailed molecular mechanisms of this diurnal regulation are unknown, while eukaryotic protein translation is mainly controlled at the level of ribosomal initiation complexes, which also involves multiple events of protein phosphorylation. We characterized the extent of protein phosphorylation in cytosolic ribosomes isolated from leaves of the model plant *Arabidopsis thaliana* harvested during day or night. Proteomic analyses of preparations corresponding to both phases of the photoperiod detected phosphorylation at eight serine residues in the C-termini of six ribosomal proteins: S2-3, S6-1, S6-2, P0-2, P1 and L29-1. This included previously unknown phosphorylation of the 40S ribosomal protein S6 at Ser-231. Relative quantification of the phosphorylated peptides using stable isotope labeling and mass spectrometry revealed a 2.2 times increase in the day/night phosphorylation ratio at this site. Phosphorylation of the S6-1 and S6-2 variants of the same protein at Ser-240 increased by the factors of 4.2 and 1.8, respectively. The 1.6 increase in phosphorylation during the day was also found at Ser-58 of the 60S ribosomal protein L29-1. It is suggested that differential phosphorylation of the ribosomal proteins S6-1, S6-2 and L29-1 may contribute to modulation of the diurnal protein synthesis in plants.

## Introduction

Living organisms coordinate biochemical, physiological and behavioral processes with alternating day and night cycles and respond to the daily oscillations in environmental conditions by specific adjustment in their metabolism and growth. In plants, due to their sessile nature, extensive circadian clock networks regulate almost every biological process, critically affecting plant fitness and adaptation [1]. The daily alternations between light and darkness cause massive changes in the carbon budget of leaves with the complex relationships between transcript levels, enzyme activities, and diurnal metabolism of starch [2]. During the day phase of photoperiod translation rates for numerous proteins and central metabolic enzymes are increased. In the model plant *Arabidopsis thaliana* the estimated rates of protein synthesis are 50–150% higher in the light phase of the photoperiod, which correlates with 50–100% increase in the activities of the key enzymes involved in the light-stimulated metabolism [3]. Measurements of distribution of ribosomes between the free and polysomal fractions in the same study indicated that protein synthesis was about twofold lower in the dark period than in the light period. Decrease in the ribosomal occupancy of transcripts had also been observed in the plant leaves during nights [3]. However, the molecular mechanisms modulating changes in the steady state of plant protein synthesis during day and night cycles are poorly understood.

The eukaryotic protein translation is mainly controlled at the level of initiation, which involves multiple events of protein phosphorylation [4]. In higher plants the changes in phosphorylation status of ribosomal protein S6 were found responsible for rapid adjustments in their growth patterns under environmental changes [5]. Accumulation of hyper-phosphorylated isoforms of the S6 protein was found elevated in root tips of maize in conditions of cold stress, while it has been reduced in response to oxygen deprivation and heat shock [6]. Arrest in translation initiation of photosynthetic transcripts at 80S cytoplasmic ribosomes caused by singlet oxygen production induced in barely correlated with a decline in the phosphorylation level of the ribosomal protein S6 [7]. A plant hormone auxin, known as a stimulator of protein synthesis in many plant tissues [8], enhanced S6 protein phosphorylation on the 40S ribosomal subunit in maize embryonic axes in line with selectively increased ribosomal protein synthesis [9]. Application of okadaic acid or heat shock to maize axes in the same study established a positive correlation between the levels of S6 phosphorylation and the ribosomal protein transcript recruitment into polysomes. The reversible decline in phosphorylation level of S6 in response to heat shock was observed in tomato cell cultures in the earlier investigation [10]. Phytohormone-induced S6 phosphorylation and translational up-regulation of ribosomal proteins and S18A mRNAs had been also described in *Arabidopsis* [11].

Located in the mRNA binding site of the 40S subunit, ribosomal protein S6 undergoes C-terminal phosphorylation in response to mitogenic stimuli in all eukaryotic cells [12]. However, it is important to stress that C-termini of the S6 proteins in plants and animals do not have any significant sequence similarity [6], [12]. Mapping of several phosphorylation sites in the ribosomal protein S6 from *Arabidopsis* and maize has been reported. Phosphorylation of Ser-240 was identified in two of S6 isoforms in *Arabidopsis* cell culture [13]. The large-scale phosphoproteome study of *Arabidopsis* seedlings characterized phosphorylation of Ser-127, Ser-237, Ser-240, Ser-247, Thr-249 in the S6-1 isoform and phosphorylation of Ser-137, Ser-240 in the S6-2 isoform [14]. Multiple phosphorylations of the two isoforms of S6 in maize roots had also been characterized [6]. Five potential phosphorylation sites in the C-termini of the maize S6 protein isoforms were suggested with localization of at least two phosphorylated residues corresponding to the positions of Ser-237 and Ser-240 in the sequence of *Arabidopsis* proteins [6]. In addition to the phosphoprotein S6, acidic P-proteins of plant ribosomes also undergo C-terminal phosphorylation which could play a role in translational responses to external stimuli. In *Arabidopsis* phosphorylation of Ser-103 in the 60S acidic ribosomal protein P1 (isoforms P1-1, P1-2, P1-3) and of Ser-305 in the 60S acidic ribosomal protein P0-2 had been detected [14]. The P-proteins form a lateral stalk structure in the active site of the 60S ribosomal subunit and are thought to be involved in regulation of translation via interaction with elongation factors [15].

In this work, we characterized the extent of protein phosphorylation in cytosolic ribosomes isolated from leaves of the model plant *Arabidopsis thaliana* harvested during day or night. Proteomic analyses of ribosomal preparations corresponding to both phases of the photoperiod detected phosphorylation at eight serine residues in the C-termini of six ribosomal proteins, including previously unknown phosphorylation of the 40S ribosomal protein S6 at Ser-231. Relative quantification of the phosphorylated peptides using stable isotope labeling and mass spectrometry revealed increase in the day/night phosphorylation ratio at this site, as well as at three other sites in the ribosomal proteins S6-1, S6-2 and L29-1, suggesting that their differential phosphorylation may contribute to modulation of the diurnal protein synthesis.

## Results

### Isolation of ribosomal proteins from the leaves of *Arabidopsis thaliana*

To characterize ribosomal protein phosphorylation in the leaves of *Arabidopsis thaliana* harvested during day or night we employed the method that previously has been used for isolation of ribosomal proteins from maize roots [6]. The method is based on solubilization of the plant cells by the mixture of four detergents, Triton X-100, Brij 35, Tween-40 and NP40, and following ultracentrifugation of ribosomal particles through sucrose cushion. The optimized procedure, described in Materials and Methods, allowed preparation of 200 to 250 µg of ribosomal proteins from 10 g of *Arabidopsis* leaves. Plants were grown under a short day cycle: 8 hours of light and 16 hours of darkness. Leaves were harvested ether in the middle of the day (4 hours of light) or 2 hours before the end of the night (14 hours of darkness). According to the study of photoperiodic protein synthesis in *Arabidopsis* [3] the selected time points corresponded well to the translational steady states of the light and dark phases of photoperiod. In total we made four independent ribosomal preparations from the leaves harvested during four different days and four preparations from the leaves harvested during four different nights. The absorbance ratio A260/A280 in all of these preparations was between 2.0 and 2.1. The protein composition of every preparation was analyzed by nano-LC-MS/MS, described below. To protect the natural phosphorylation state of ribosomal proteins at all stages of preparations we used two phosphatase inhibitors, sodium molybdate and β-glycerophosphate, according to the earlier described procedure [6].

### Characterization of the ribosomal proteins by nano-LC-MS/MS

**Figure 1:**
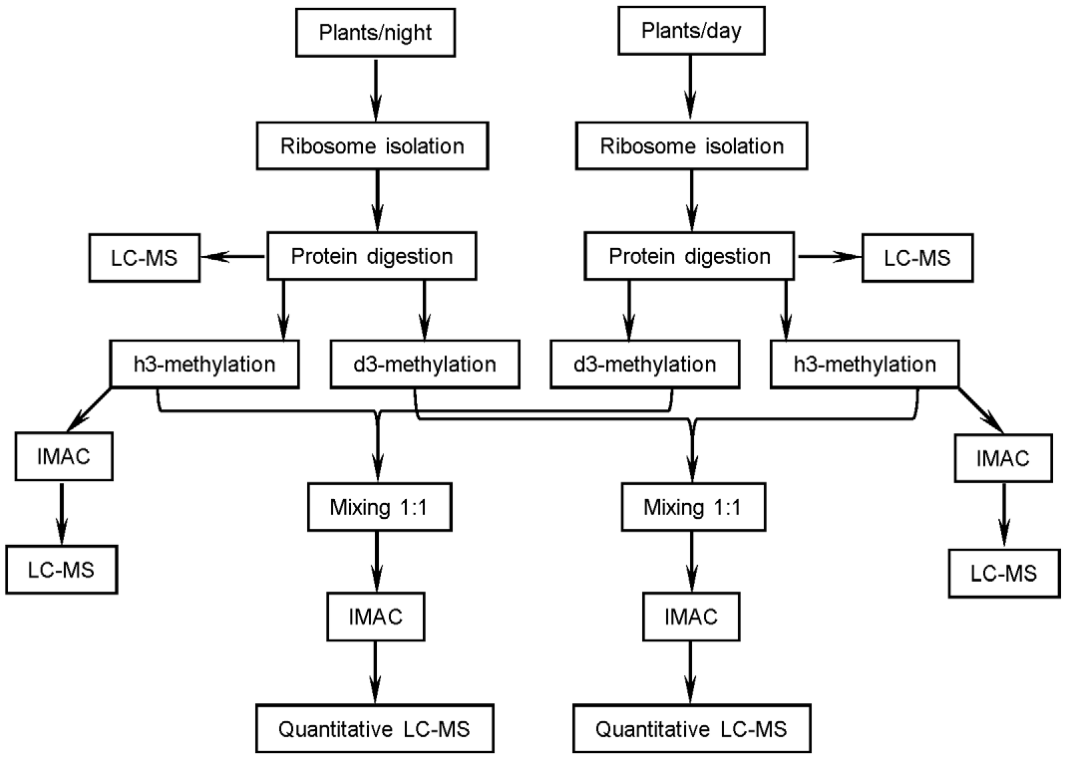
A scheme for preparation and analyses of ribosomal proteins from leaves of *Arabisopsis thaliana* harvested during day or night.

For proteomic characterization an aliquot from every ribosomal preparation was digested by trypsin and 1 to 2 µg of digested protein samples were analyzed by nano-LC-MS/MS. Peptide ions were sequenced using two alternating fragmentation techniques: collision induced dissociation (CID) and electron transfer dissociation (ETD). The data obtained after CID and ETD peptide sequencing were separately analyzed using MASCOT search against the Swissprot database for *Arabidopsis*. Then we made combined analysis of the data collecting in either eight CID or eight ETD sequencing data sets using the Scaffold proteomics program. The resulting set of identified cytosolic ribosomal proteins is shown in Table S1. We were able to detect 73 of 80 protein families composing the cytosolic ribosome in *Arabidopsis* [13]. Table S1 summarizes data on a number of sequenced and unique peptides identified in 95 cytosolic ribosomal proteins with indicated gene locus. Out of these proteins 40 were from the 40S ribosomal subunit and 55 were from the 60S ribosomal subunit. The chloroplastic ribosome from the same preparations was represented by 28 proteins: 12 from 30S and 26 from 50S ribosomal subunits (not shown). Statistical analysis embedded in the Scaffold proteomics program did not detect significant difference in protein composition between the “day” preparations on the one hand and the “night” preparations on the other hand. At the following step we used Scaffold's “Quantify” analysis of spectral count numbers for every protein in each biological sample and selected four out of eight ribosomal preparations for further comparative characterization. This selection was done on the basis of similarity in amount of ribosomal proteins and included two biological replicates corresponding to “night” and two biological replicates corresponding to “day” (Table S2). For comparative analysis of protein phosphorylation in these samples we used the strategy schematically outlined in Figure 1. The protein phosphorylation analyses included two sequential steps: (i) mapping of phosphorylation sites in the ribosomal proteins, and (ii) relative quantification of protein phosphorylation using differential stable isotope labeling.

### Mapping of phosphorylation sites in the ribosomal proteins

To map phosphorylation sites in the ribosomal proteins we used the immobilized metal affinity chromatography (IMAC) procedure [16], [17], [18]. Phosphorylated peptides enriched by IMAC were further separated using reverse-phase nano-LC and sequenced by alternating CID and ETD MS/MS. The data were analyzed using the MASCOT search engine as well as by manual verification of CID and ETD MS/MS spectra of each phosphorylated peptide. This analysis revealed eight phosphoserine residues in the C-termini of six ribosomal proteins (Table 1, Figure S1). All of these phosphorylated peptides were found in the ribosomal preparation from both phases of photoperiod. Seven phosphorylated peptides have been sequenced in the earlier studies [13], [14]: phosphopeptide KDEPAEEs^103^DGDLGFGLFD from the 60S acidic ribosomal protein P1, which is identical in its P1-1, P1-2 and P1-3 isoforms; VEEKEEs^305^DEEDYG from the 60S acidic ribosomal protein P0-2; ALs^273^TSKPDPVVEDQA from the 40S ribosomal protein S2-3; AGENAs^58^AEE from the 60S ribosomal protein L29-1; s^237^RLs^240^SAAAKPSVTA from the 40S ribosomal protein S6-1 and s^237^RLs^240^SAPAKPVAA from the 40S ribosomal protein S6-2 (Table 1). Additionally we identified 40S ribosomal protein S6 phosphorylation at Ser-231, which has not been previously characterized.

**Table 1:** Ribosomal phosphopeptides identified using LC-MS/MS after IMAC.

Protein isoforms, gene locus	Peptide sequence	M/Z	Z	Score/E-value
**60S ribosomal protein P1**	KDEPAEEs^103^DGDLGFGLFD	1066.9	2+	92/3.6e-08
(P1-1, P1-2, P1-3)				
At1 g01100, At4 g00810,				
At5 g47700				
**40S ribosomal protein S6**	SEs^231^LAK	371.6	2+	20/0.4
		742.3	1+	26/0.14
	RSEs^231^LAK*	449.7	2+	44/0.0014
S6-2, At5 g10360	SRLs^240^SAPAKPVAA	674.8	2+	59/0.0024
	s^237^RLs^240^SAPAKPVAA	714.7	2+	41/0.19
S6-1, At4 g31700	SRLs^240^SAAAK	492.7	2+	57/0.0032
	SRLs^240^SAAAKPSVTA	720.3	2+	59/5.1e-05
	s^237^RLs^240^SAAAKPSVTA	760.3	2+	37/0.0075
**60S ribosomal protein P0-2**	VEEKEEs^305^DEEDYG	882.3	2+	54/0.0084
At3 g09200				
**40S ribosomal protein S2-3**	ALs^273^TSKPDPVVEDQA	846.8	2+	57/0.004
At2 g41840				
**60S ribosomal protein L29-1**	AGENAs^58^AEE	507.1	2+	47/0.042
At3 g06700		1013.3	1+	30/1.9

M/Z - mass over charge ratio; Z – ion charge. The experimentally found peptide masses correspond to the masses of phosphopeptides with methyl-esterified dicarboxylic and carboxyl-terminal amino acid residues. Lowercase s in the sequences designate phosphorylated serine residues with the superscript numbers corresponding to the amino acid positions.

*Peptide RSEs^231^LAK with one missed cleavage was obtained in separate experiment of limited proteolysis when ribosomal proteins were treated with trypsin for only two hours at 37 °C.

**Figure 2:**
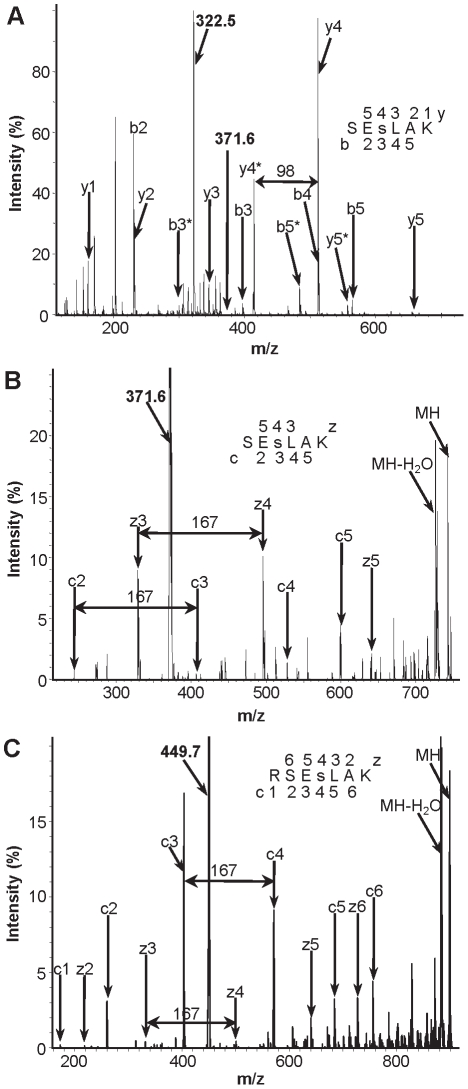
Identification of Ser-231 phosphorylation in the 40S ribosomal protein S6 by mass spectrometric sequencing of two phosphorylated peptides. **A**, CID spectrum of methylated doubly charged phosphopeptide SEs^231^LAK with mass over charge ratio (M/Z) 371.6, as indicated. The doubly charged ion indicated at M/Z = 322.5 corresponds to the parent ion after the neutral loss of phosphoric acid (H_3_PO_4_; 98 Da). The b (N-terminal) and y (C-terminal) fragment ions are labeled in the spectrum and in the displayed peptide sequence. Lowercase s in the sequence designates phosphorylated serine residue. Phosphorylation site was localized according to the pattern of the fragment ions containing phosphate and corresponding fragments with the neutral loss, which are marked with asterisks. Phosphorylation of the serine residue is evident from the distinct set of the fragments: y4, y4*, and y3, b2, b3, b3*. B, ETD spectrum of the same peptide. The c and z fragment ions are labeled in the spectrum. The indicated mass increment of 167 between z3 and z4, as well as between c2 and c3 corresponds to the intact phosphorylated serine residue at the third position from the peptide N-terminus. C, ETD spectrum of the doubly charged methylated peptide RSEs^231^LAK with one missed cleavage and M/Z = 449.7 (indicated). The c and z fragment ions are labeled in the spectrum. The indicated mass increment of 167 between z3 and z4, as well as between c3 and c4 corresponds to the intact phosphorylated serine residue typed as the lowercase s in the shown peptide sequence.

We detected a novel phosphorylated peptide SEs^231^LAK, which can originate from both isoforms of the 40S ribosomal protein S6 (Table 1). The phosphorylation site in this peptide was assigned to the third amino acid residue from the N-terminus, corresponding to Ser-231 in the protein sequence. This localization of the phosphorylation site was determined using both CID (Figure 2A) and ETD (Figure 2B) sequencing. If in case of the CID fragmentation this phosphorylated residue produced a neutral loss signal of phosphoric acid (see for example y4 and y4* fragments in Figure 2A), ETD sequencing revealed the signals corresponding to intact phosphoserine (indicated as fragment mass difference of 167 in Figure 2B). However, because of the short length of this peptide the MASCOT ion score and expect value parameters assigned to this peptide in the database search were rather low (Table 1). To get the additional confirmation of this phosphorylation we made a separate experiment of limited proteolysis when ribosomal proteins were treated with trypsin for only two hours. This treatment produced a peptide RSEs^231^LAK with one missed cleavage and more solid MASCOT identification parameters (Table 1, Figure S1). The ETD fragmentation spectrum of this peptide is shown in the Figure 2C.

### Relative quantification of protein phosphorylation using differential stable isotope labeling

All phosphopeptides listed in Table 1 were found in the protein samples obtained from the plant leaves harvested during both day and night. To check if the level of this ribosomal protein phosphorylation is diurnaly regulated we made comparative quantification using differential stable isotope labeling and mass spectrometry. The workflow for these experiments is outlined in Figure 1. Carboxyl groups of the tryptic ribosomal peptides isolated from leaves harvested during either day or night were esterified with anhydrous h3-methyl alcohol or d3-methyl d-alcohol [16], [17], [18]. A mass increment of 14 Da or 17 Da per each esterified carboxyl group was obtained in the peptides modified by either h3-methanol or d3-methanol, correspondingly. Light-isotope labeled peptides from plants harvested during day were mixed in the ratio 1∶1 (on the bases of the same protein amount) with heavy-isotope labeled peptides from plants harvested during night. The reversed labeling of the same peptide samples has been done as a separate experiment and an internal control. The methyl esters of the differentially labeled phosphorylated peptide mixtures were then enriched by IMAC and analyzed by nano-LC-MS. At the latter stage in every nano-LC-MS run we determined intensities of the phosphopeptide pairs labeled with light- and heavy-isotopes. The quantification of the differentially labeled signals determined relative change in the extent of phosphorylation for each individual ribosomal peptide. The control reversed labeling experiment had to produce the same phosphorylation ratio for every particular peptide from the samples corresponding to the day and night phases of photoperiod.

**Figure 3:**
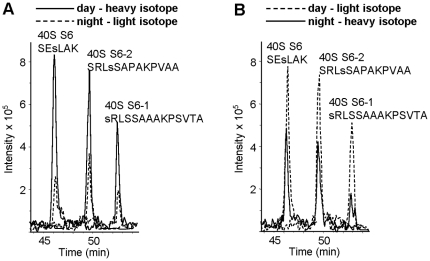
Quantitative mass spectrometric analysis of phosphorylated peptides from the 40S ribosomal protein S6. Ion chromatograms of three phosphorylated peptides differentially labeled with light or heavy isotopes and analyzed by nano-LC-MS. **A**, ion intensity peaks indicated with continuous line correspond to peptides SEs^231^LAK with M/Z = 748.3, SRLs^240^SAPAKPVAA with M/Z = 676.3 and SRLs^240^SAAAKPSVTA with M/Z = 721.8 from plants harvested during day time and labeled by heavy, d3-methanol. Ion intensity peaks indicated with dashed line correspond to the same peptides obtained from the plants harvested during night and labeled by light h3-methanol. M/Z values for the light isotope labeled phosphopeptides are 742.3, 674.8 and 720.3, correspondingly. The sequences of the phosphorylated peptides from S6 are shown above the corresponding peaks. **B**, ion intensity peaks as in A except that the peptides from the plants harvested during day were labeled with light isotope (dashed line) and the peptides from the plants harvested during night were labeled with heavy isotope (continuous line).

**Figure 4:**
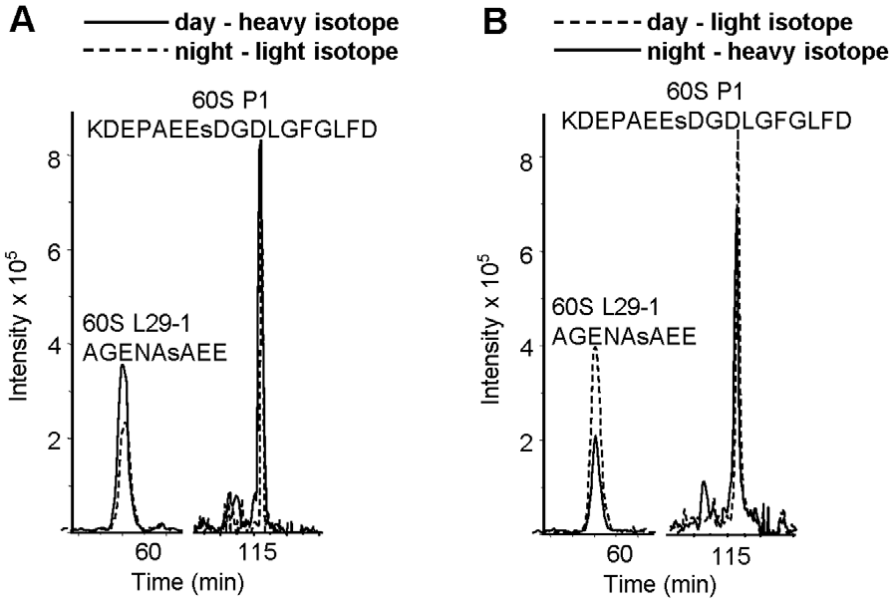
Quantitative mass spectrometric analysis of phosphorylated peptides from the 60S ribosomal protein L29-1 and from the 60S acidic ribosomal protein P1. **A**, ion chromatograms of phosphorylated peptides from the plants harvested during day labeled with heavy isotope (continuous line) and from the plants harvested during night labeled with light isotope (dashed line). The sequences of two phosphorylated peptides: AGENAs^58^AEE (M/Z = 1025.3 for heavy and M/Z = 1013.3 for light isotope labeled peptide) from the 60S ribosomal protein L29-1 and KDEPAEEs^103^DGDLGFGLFD (M/Z = 1078.9 for heavy and M/Z = 1066.9 for light isotope labeled peptide) from the 60S acidic ribosomal protein P1 are shown above the corresponding ion intensity peaks. B, ion intensity peaks as in A except that peptides from the plants harvested during day were labeled with light isotope (dashed line) and peptides from the plants harvested at night with heavy isotope (continuous line).

The differential labeling technique allowed reliable quantification of three phosphorylated peptides from the 40S ribosomal protein S6 (Figure 3). In the case of the phosphorylated peptide SEs^231^LAK, found in the present study for the first time, its mass over charge ratio (M/Z) was either 742.3 or 748.3 when it was labeled with h3-methanol or d3-methanol, correspondingly. Solid line in Figure 3A shows that intensity of the 748.3 signal from the “day” sample, which is more than two times higher than intensity of the 742.3 signal from the “night” sample. In the reciprocal labeling experiment (Figure 3B) the “day” sample is represented by the light isotope labeled peptide (742.3, dashed line) which intensity is still twice higher than the signal from the “night” sample. Two other phosphorylated peptides from this protein represented phosphorylation of Ser-240 in S6-1 and S6-2 isoforms. Differential labeling of these peptides demonstrated that phosphorylation of Ser-240 in S6-1 and S6-2 was 4 and 2 times higher in the samples from the leaves harvested during the day (Figure 3). We also observed reproducible day-time increase in phosphorylation of Ser-103 in the 60S acidic ribosomal protein P1 and of Ser-58 in the 60S ribosomal protein L29-1 (Figure 4). Quantitative data estimated 1.3 and 1.6 day-time increase in phosphorylation of these two residues (Table 2). The day to night phosphorylation ratio of three serine residues in the S6 protein was in the range between 1.8 and 4.2 (Table 2). In case of the doubly phosphorylated peptides from S6-1 and S6-2, as well as phosphorylated peptides from the 60S ribosomal protein P0-2 and the 40S ribosomal protein S2-3 (Table 1) we were not able to get a reliable quantitative data because of the low signal to noise ratios. However, the data presented in Table 2 revealed that phosphorylation of three ribosomal proteins at four different serine residues was increased during the day time.

**Table 2:** Relative quantitation of ribosomal phosphopeptides isolated from leaves harvested at day or night.

Protein isoforms, gene locus	Peptide sequence	Phosphorylation ratio day/night
**60S acidic ribosomal protein P1**	KDEPAEEs^103^DGDLGFGLFD	1.3±0.3
(P1-1, P1-2, P1-3) At1 g01100, At4 g00810, At5 g47700		
**40S ribosomal protein S6**	SEs^231^LAK	2.2±0.2**
S6-2, At5 g10360	SRLs^240^SAPAKPVAA	1.8±0.2**
S6-1, At4 g31700	SRLs^240^SAAAKPSVTA	4.2±0.4**
**60S ribosomal protein L29-1** At3 g06700	AGENAs^58^AEE	1.6±0.3*

Lowercase s in the sequences designate phosphorylated serine residue with the superscript numbers corresponding to the amino acid position in the protein. The data are averages (+/- standard deviation) from four LC-MS runs corresponding to the two day and two night preparations (Table S2) and two types of labeling for each day/night pair (see Figure 1). * and ** represent statistically significant increase at *P* <0.05 and *P* <0.01, respectively, by Student's *t* test.

## Discussion

In this work we addressed the question of qualitative and quantitative changes in phosphorylation of ribosomal proteins from *Arabidopsis* plants harvested during night or day. For isolation of the ribosomal proteins we adapted the earlier described protocol [6], which yielded preparations enriched in cytosolic ribosomes. The earlier study [3] demonstrated that the amount of cytosolic ribosomes in *Arabidopsis* leaves is significantly higher than that of the plastid and mitochondrial ribosomes. In accordance with this in each of our preparations we were able to identify 95 cytosolic ribosomal proteins (Table S1). Almost identical protein compositions in the samples from the leaves harvested during day or night was confirmed using Scaffold proteomic program. Similarity of these preparations allowed us to make comparative analysis of protein phosphorylation. In all preparations we found phosphorylation of six ribosomal proteins (the 60S acidic ribosomal protein P1, the 60S acidic ribosomal protein P0-2; the 40S ribosomal protein S2-3; the 60S ribosomal protein L29-1; the 40S ribosomal protein S6-1 and the 40S ribosomal protein S6-2) at eight different phosphorylation sites. This finding demonstrated absence of qualitative difference in the diurnal protein phosphorylation. However, application of the stable isotope labeling technique revealed the quantitative increase in the day-to-night ratio of phosphorylation of the 40S ribosomal protein S6-1, the 40S ribosomal protein S6-2 and the 60S ribosomal protein L29-1.

**Figure 5:**
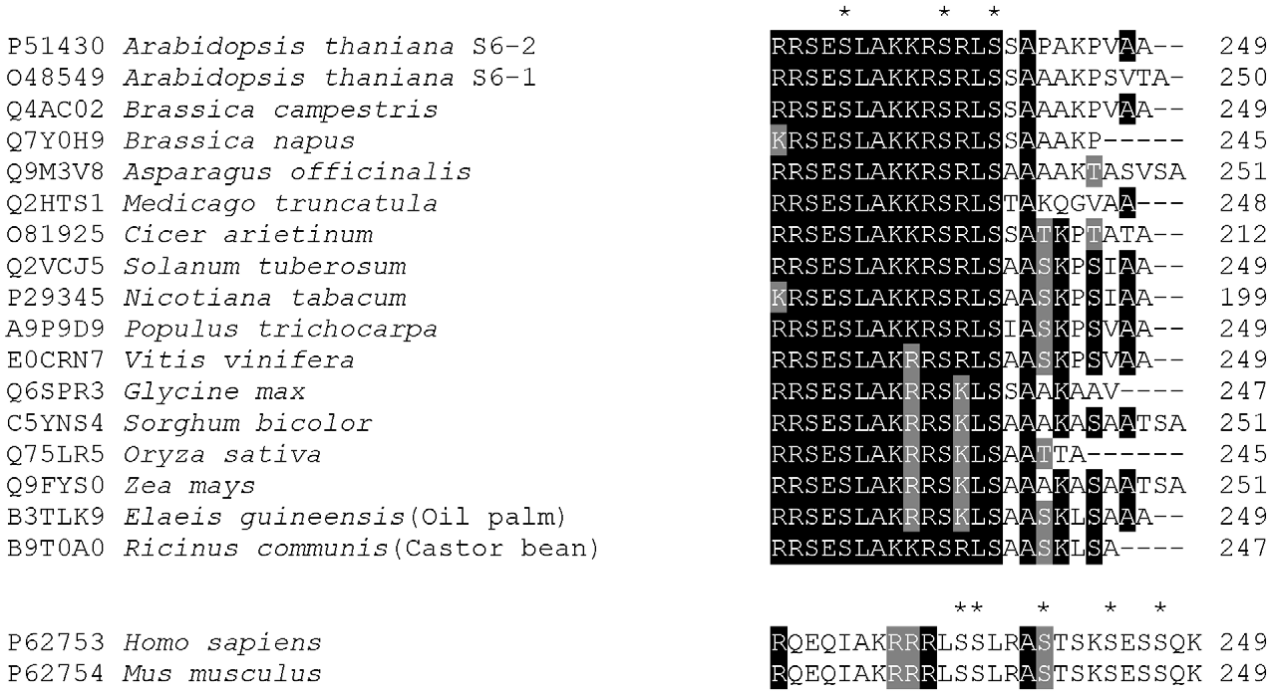
Conservation of three phosphorylated serine residues in C-termini of the 40S ribosomal proteins S6 from higher plants. Alignment of C-terminal sequences from the 40S ribosomal proteins S6 of higher plants. UniProt accession numbers, organism names and numbers of the last amino acids in corresponding protein sequences are indicated. Positions of three serine residues found phosphorylated in S6-1 and S6-2 proteins from *Arabidopsis thaliana* are indicated by asterisks. Below the alignment the two identical C-terminal sequences from the 40S ribosomal proteins S6 of *Homo sapiens* and *Mus musculus* are shown. Positions of five conserved serine residues phosphorylated in animal S6 proteins are indicated by asterisks.

Importantly, we identified a previously unknown phosphorylation site Ser-231 in the 40S ribosomal protein S6. Mapping of this novel phosphorylation site was achieved mainly because of the use of the relatively new ETD sequencing technique, which is soft in comparison with CID fragmentation used in previous studies [13], [14]. CID causes significant distraction of phosphorylated residues with concomitant production of neutral loss signals in the tandem mass spectra (see Figure 2A), while ETD detects intact phosphorylated residues (see Figure 2B,C). Recent parallel analyses of different peptide mixtures by ETD and CID sequencing demonstrated significantly higher rate of phosphopeptide detection by ETD [19], [20]. Moreover, ETD allows for mapping of the exact phosphorylation sites in the peptides containing several residues susceptible to this modification [21]. Comparative analysis of the C-terminal sequences of the S6 proteins from higher plants demonstrates that Ser-231, as well as Ser-237 and Ser-240 are highly conserved (Figure 5). The conservation of these three residues found to be phosphorylated in our study is quite remarkable in comparison with the other serine or threonine residues proposed as additional phosphorylation sites in the C-termini of the S6 protein from *Arabidopsis* and maize [6], [14].

Ribosomal protein S6 undergoes C-terminal phosphorylation in response to mitogenic stimuli in all eukaryotic cells [12], however, there is a significant difference in localization of the phosphorylated residues between different species. Phosphorylation of the *Saccharomyces cerevisiae* ribosomal protein S6 proceeds at two serine residues [22] while there are five different phosphorylation sites in S6 from animals [12], [23]. Mapping of phosphorylation sites in the plant proteins made in the present and previous studies [6], [14] reveals significant difference from positions of phosphorylated residues in animal S6. Below the alignment of the C-termini from plant ribosomal protein S6 in Figure 5 we show the corresponding sequences from human and mouse proteins. Because of the low sequence similarity between the animal and plant proteins we align three consecutive positively charged amino acid residues (RRR in animal proteins and KKR in plant proteins). These three residues in the S6 ribosomal protein from rat had been demonstrated to be curtail substrate recognition cluster for mitogen-activated 70 K S6 kinase [23]. The absence of conservation for serine residues in the S6 proteins suggests different potential mechanisms for functional modulation of this protein by phosphorylation in animals and plants. In search for the physiological function of this phosphorylation a knock-in mouse encoding a mutant rpS6 harboring Ala substitutions at all five C-terminal phosphorylation sites (see Figure 5) had been constructed and intensively studied [24]. These mutations resulted in reduced overall size, glucose intolerance, and muscle weakness, as well as reduced cell size, however, overall protein translation was not grossly reduced in rpS6 knock-in cells [24]. The complexity of these regulatory effects is enhanced by findings of multiple protein kinases involved in differential phosphorylation of these serine residues [25]. In any case, conservation of plant-specific phosphorylation sites (Figure 5) and pronounced modulation of plant S6 protein phosphorylation by phytohormones [9], [11] suggest specific physiological role for C-terminal modifications of this ribosomal protein in higher plants.

The previous studies on different plants and plant cells established positive correlation between phosphorylation of the S6 protein and translation, particularly synthesis of ribosomal proteins [5], [6], [7], [9], [10], [11]. We found 2 to 4 fold increase in phosphorylation of tree C-terminal residues of S6 ribosomal protein isolated from *Arabidopsis* leaves harvested during day time, as compared to the protein from the leaves harvested at night. The higher phosphorylation of S6 could be at least partially responsible for the elevated level of general protein synthesis in the day time of the photoperiod [3]. Additionally we found moderate “day time” increase in phosphorylation of the 60S ribosomal proteins L29-1. The exact mechanisms for increase of protein synthesis in the light phase of photoperiod are still unknown, while our findings suggest a possibility of diurnal regulation of translation in plants by differential combinatorial phosphorylation of at least three ribosomal proteins: the 40S ribosomal proteins S6-1 and S6-2, and the 60S ribosomal protein L29-1.

## Materials and Methods

### Plant material and growth conditions

*Arabidopsis thaliana* wild type (ecotype Columbia 0) plants were grown hydroponically [26] at 20–22 °C and 65–70% relative humidity under a 8 h/16 h light/dark photoperiod at light intensity of 150 µmol of photons m^−2^s^−1^.

### Isolation of ribosomes from the plant leaves

Plant leaves were harvested either after light adaption (4 h after the light period started) or after dark adaption (2 hours before the dark period ended). Ribosomes were isolated essentially as described in [6]. The plant material (10 g) was ground in liquid nitrogen with a mortar and pestle, incubated in 20 mL of the ice cold ribosome extraction buffer (200 mM Tris-HCl pH 7.5, 200 mM KCl, 25 mM EGTA, 36 mM MgCl_2_, 1 mM sodium molybdate, 1 mM dithiothreitol, 50 µg mL^−1^ cyclohexamide, 50 µg mL^−1^ chloramphenicol, 80 mM β-glycerophosphate, 1% [v/v] Triton X-100, 1% [v/v] Brij 35, 1% [v/v] Tween-40 and 1% [v/v] NP40) with magnetic stirring for 20 min at 4 °C and filtered through four layers of nylon mesh (20 µm pore size). Cell debris was removed by centrifugation two times at 10 000 g for 15 min at 4 °C. The supernatant was layered on top of a sucrose cushion (1.3 M sucrose, 400 mM Tris-HCl pH 7.5, 200 mM KCl, 5 mM EGTA, 36 mM MgCl_2_, 1 mM sodium molybdate, 1 mM dithiothreitol, 50 µg mL^−1^ cyclohexamide, 50 µg mL^−1^ chloramphenicol, 80 mM β-glycerophophate) and the ribosomes were pelleted by centrifugation at 149 000 g for 18 h at 4 °C. The resulting pellet was carefully resuspended by rotation for 2 h at 4 °C in Staehlin A buffer (20 mM Tris-HCl pH 7.5, 5 mM MgCl_2_, 1 mM sodium molybdate and 1 mM dithiothreitol). The sample was centrifuged at 14 000 g for 15 min at 4 °C to pellet insoluble material. Absorbance readings of A260 and A280 were conducted to quantify the ribosomes (A260/11.1 corresponds to mg/ml of ribosomes). The sample was diluted in 0.1 volume 1 M MgCl_2_ and 2 volumes glacial acetic acid, vortexed for 1 h at 4 °C and centrifuged at 14 000 g for 10 min at 4 °C to remove RNA. Proteins were precipitated by adding 5 volumes of acetone, centrifuged at 14 000 g for 5 min at 4 °C, washed with two changes of acetone and then with two changes of ethanol for 60 min and air dried.

### Protein identification and peptide analyses by LC-MS/MS

The ribosomal protein pellet was resuspended in 50 mM NH_4_HCO_3_ and protein concentration was measured according to Bradford [27]. The cystein residues were reduced with 2 mM DTT for 1 h at 50 °C and alkylated by 6 mM iodoacetamide for 1 h at room temperature in darkness. Trypsin (Sequencing Grade Modified, Promega) was added to final concentration 0.03 mg trypsin/mg protein. Digestions were performed for 20 h at 37 °C and obtained peptide mixtures were analyzed by LC-MS/MS. Separation was done using nano-flow HPLC system (EASY-nLC fromBruker Daltonics, Bremen, Germany) and data were acquired using on-line electrospray ionization ion trap “HCTultra PTM Discovery System” (Bruker Daltonics, Bremen, Germany). Peptides were separated by reverse phase chromatography on a 20 mm×100 µm (particle size 5 µm) C18 pre column followed by a 100 mm×75 µm C18 column (particle size 5 µm) at a flow rate 300 nL/min. A gradient of 0.1% formic acid in water (A) and 0.1% formic acid in acetonitrile (B) was distributed as follows: starting with 0% B; linear gradient 0%−20% B in 0–120 min; 20%–30% B in 120–150 min; 30%–100% B in 150–170 min; 100% B in 170–180 min.

The automated online tandem MS analyses were performed using alternating collision induced dissociation (CID) and electron transfer dissociation (ETD) of peptide ions. A full-scan MS acquisition was performed in "standard-enhanced" mode (8 100 m/z/s) followed by two MS/MS scans in "ultra scan" mode (26 000 m/z/s) on the five most abundant ions. LC-MS/MS analysis was made at least two times for each sample. Peak lists were created from the raw data using Bruker Daltonics DataAnalysis 3.4 (Bruker Daltonics, Bremen, Germany) and the resulting MGF files were used to search for *Arabidopsis thaliana* proteins in Swissprot (UniProtKB/Swiss-Prot Release 2011_03) on an in-house Mascot server (version 2.2.06) (www.matrixscience.com). The search parameters allowed mass errors up to 0.6 Da for MS data, and up to 0.6 Da for MS/MS data in case of CID of peptide ions and 1.5 Da in case of ETD of peptide ions. The charge states of the peptides were varied; three missed cleavage sites were permitted. Cysteine carbamidomethylation was selected as a fixed modification. N-terminal protein acetylation and serine, threonine or tyrosine phosphorylation were selected as variable modifications. For phosphopeptides enriched with IMAC the methylation of aspartic and glutamic acids, as well as methylation of C-terminal carboxylic groups were additionally selected as fixed modifications. The identifications of peptides were considered reliable if they were sequenced by both CID and ETD techniques and the MS/MS spectra had MASCOT scores above 25. The sequence of each phosphorylated peptide was verified by manual analysis of MS/MS spectra. A combined analysis of MASCOT-search results collecting in either CID or ETD sequencing data sets for samples corresponding to each phase of photoperiod was performed using Scaffold (version Scaffold_3.0.00, Proteome Software Inc., Portland, OR) proteomics program to select ribosomal proteins identified in all preparations.

### Identification and relative quantification of phosphorylated peptides

The ribosomal proteins digested by trypsin for 20 h or 2 h (limited proteolysis) were vacuum dried, methylesterified by methanolic HCl [16] and enriched for phosphorylated peptides by the IMAC procedure described in [16], [17], [18]. The quantification of protein phosphorylation was performed as described in [18]. Vacuum dried peptides from 50 µg of protein from the leaves harvested during either day or night were methyl-esterified with either 200 µL of 2 N methanolic HCl or DCl prepared by addition of 160 µL of acetylchloride to 1 mL of anhydrous h3-methyl alcohol or to 1 mL of anhydrous d3-methyl d-alcohol (Sigma-Aldrich). The reactions were performed at room temperature for 2 h. The peptides were lyophilized and esterification procedure was repeated once more. Light- and heavy-isotope labeled peptides were mixed 1∶1 and enriched for phosphopeptides by the IMAC method described in [18]. The reverse labeling of the peptides from the leaves harvested during either day or night was performed as an internal control. Peptide mixtures were analyzed by LC-MS/MS. For the comparative quantification of differentially labeled phosphorylated peptides, the extracted ion chromatograms acquired using LC-MS were computed for the selected peptide ions. The extracted ion chromatogram peak area represented the total ion abundance of the selected peptide. Analyses were performed using Bruker Daltonics DataAnalysis 3.4 (Bruker Daltonics, Bremen, Germany). The peptide sequence alignments were made using ClustalW2 and Boxshade programs and shading represents the degree of conservation at each position, taking into account similar physicochemical properties of the residues.

## Supporting Information

Figure S1 **Peptide identification views from MASCOT MS data analyses of phosphorylated peptides sequenced by collision induced dissociation (CID) and electron transfer dissociation (ETD) of their ions.** The spectra and corresponding lists of singly and doubly charged fragment ions identified in the MASCOT search are shown.
(DOC)

Table S1 **Ribosomal proteins identified by nano-LC-MS/MS.** A list of cytosolic ribosomal proteins obtained using Scaffold proteomic program from combined MASCOT identifications in all ribosomal protein preparations. Gene locus, number of sequenced peptides and number of unique sequenced peptides are indicated.
(DOC)

Table S2 **Spectral counts for ribosomal proteins in four biological samples (two day and two night) calculated using Scaffold proteomic program.**
(DOC)
