# Supplementary material for: Differential Phosphorylation of Ribosomal Proteins in Arabidopsis thaliana Plants during Day and Night
Source: PLoS One. 2011 Dec 16;6(12):e29307. doi: 10.1371/journal.pone.0029307 (PMC3241707; doi:10.1371/journal.pone.0029307)
Supplement: Figure S1 — Peptide identification views from MASCOT MS data analyses of phosphorylated peptides sequenced by collision induced dissociation (CID) and electron transfer dissociation (ETD) of their ions. The spectra and corresponding lists of singly and doubly charged fragment ions identified in the MASCOT search are shown. (DOC) [file pone.0029307.s001.doc]

**Figure S1**

**CID** MS/MS Fragmentation of **KDEPAEESDGDLGFGLFD**
Found in **RLA11_ARATH**, 60S acidic ribosomal protein P1-1 OS=Arabidopsis thaliana GN=RPP1A PE=1 SV=2

Match to Query 2: 2131.805448 from(1066.910000,2+)


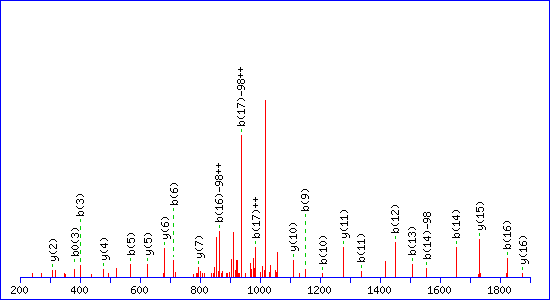


**Monoisotopic mass of neutral peptide Mr(calc):** 2131.9238

**Fixed modifications:** Methyl (C-term),Methyl (DE)

**Variable modifications:**

**S8 :** Phospho (ST), with neutral losses 0.0000(shown in table), 97.9769

**Ions Score:** 92 **Expect:** 2.6e-08

**Matches :** 24/256 fragment ions using 31 most intense peaks

| **#** | **b** | **b^++^** | **b*** | **b*^++^** | **b^0^** | **b^0++^** | **Seq.** | **y** | **y^++^** | **y^0^** | **y^0++^** | **#** |
| --- | --- | --- | --- | --- | --- | --- | --- | --- | --- | --- | --- | --- |
| **1** | 129.1022 | 65.0548 | 112.0757 | 56.5415 |  |  | **K** |  |  |  |  | **18** |
| **2** | 258.1448 | 129.5761 | 241.1183 | 121.0628 | 240.1343 | 120.5708 | **D** | 2004.8362 | 1002.9217 | 1986.8256 | 993.9164 | **17** |
| **3** | ***401.2031*** | 201.1052 | 384.1765 | 192.5919 | 383.1925 | 192.0999 | **E** | ***1875.7936*** | 938.4004 | 1857.7830 | 929.3951 | **16** |
| **4** | 498.2558 | 249.6316 | 481.2293 | 241.1183 | 480.2453 | 240.6263 | **P** | ***1732.7353*** | 866.8713 | 1714.7248 | 857.8660 | **15** |
| **5** | ***569.2930*** | 285.1501 | 552.2664 | 276.6368 | 551.2824 | 276.1448 | **A** | 1635.6826 | 818.3449 | 1617.6720 | 809.3396 | **14** |
| **6** | ***712.3512*** | 356.6792 | 695.3246 | 348.1660 | 694.3406 | 347.6740 | **E** | 1564.6455 | 782.8264 | 1546.6349 | 773.8211 | **13** |
| **7** | 855.4094 | 428.2084 | 838.3829 | 419.6951 | 837.3989 | 419.2031 | **E** | 1421.5872 | 711.2972 | 1403.5767 | 702.2920 | **12** |
| **8** | 1022.4078 | 511.7075 | 1005.3812 | 503.1943 | 1004.3972 | 502.7023 | **S** | ***1278.5290*** | 639.7681 | 1260.5184 | 630.7628 | **11** |
| **9** | ***1151.4504*** | 576.2288 | 1134.4238 | 567.7156 | 1133.4398 | 567.2236 | **D** | ***1111.5306*** | 556.2689 | 1093.5201 | 547.2637 | **10** |
| **10** | ***1208.4719*** | 604.7396 | 1191.4453 | 596.2263 | 1190.4613 | 595.7343 | **G** | 982.4880 | 491.7477 | 964.4775 | 482.7424 | **9** |
| **11** | ***1337.5144*** | 669.2609 | 1320.4879 | 660.7476 | 1319.5039 | 660.2556 | **D** | 925.4666 | 463.2369 | 907.4560 | 454.2316 | **8** |
| **12** | ***1450.5985*** | 725.8029 | 1433.5720 | 717.2896 | 1432.5879 | 716.7976 | **L** | ***796.4240*** | 398.7156 | 778.4134 | 389.7103 | **7** |
| **13** | ***1507.6200*** | 754.3136 | 1490.5934 | 745.8004 | 1489.6094 | 745.3083 | **G** | ***683.3399*** | 342.1736 | 665.3293 | 333.1683 | **6** |
| **14** | ***1654.6884*** | 827.8478 | 1637.6618 | 819.3346 | 1636.6778 | 818.8426 | **F** | ***626.3184*** | 313.6629 | 608.3079 | 304.6576 | **5** |
| **15** | 1711.7099 | 856.3586 | 1694.6833 | 847.8453 | 1693.6993 | 847.3533 | **G** | ***479.2500*** | 240.1287 | 461.2395 | 231.1234 | **4** |
| **16** | ***1824.7939*** | 912.9006 | 1807.7674 | 904.3873 | 1806.7834 | 903.8953 | **L** | 422.2286 | 211.6179 | 404.2180 | 202.6126 | **3** |
| **17** | 1971.8623 | 986.4348 | 1954.8358 | 977.9215 | 1953.8518 | 977.4295 | **F** | ***309.1445*** | 155.0759 | 291.1339 | 146.0706 | **2** |
| **18** |  |  |  |  |  |  | **D** | 162.0761 | 81.5417 | 144.0655 | 72.5364 | **1** |

**ETD** MS/MS Fragmentation of **KDEPAEESDGDLGFGLFD**
Found in **RLA11_ARATH**, 60S acidic ribosomal protein P1-1 OS=Arabidopsis thaliana GN=RPP1A PE=1 SV=2

Match to Query 2: 2131.805448 from(1066.910000,2+)


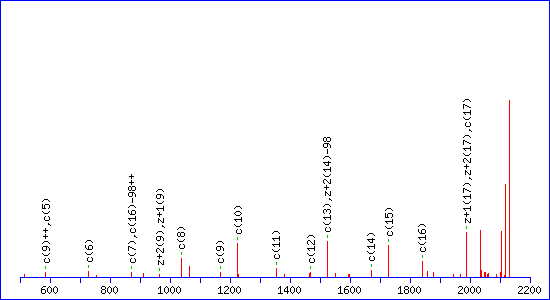


**Monoisotopic mass of neutral peptide Mr(calc):** 2131.9238

**Fixed modifications:** Methyl (C-term),Methyl (DE)

**Variable modifications:**

**S8 :** Phospho (ST), with neutral losses 0.0000(shown in table), 97.9769

**Ions Score:** 117 **Expect:** 8.3e-11

**Matches :** 20/198 fragment ions using 16 most intense peaks

| **#** | **c** | **c^++^** | **Seq.** | **y** | **y^++^** | **z+1** | **z+1^++^** | **z+2** | **z+2^++^** | **#** |
| --- | --- | --- | --- | --- | --- | --- | --- | --- | --- | --- |
| **1** | 146.1288 | 73.5680 | **K** |  |  |  |  |  |  | **18** |
| **2** | 275.1714 | 138.0893 | **D** | 2004.8362 | 1002.9217 | 1988.8175 | 994.9124 | 1989.8253 | 995.4163 | **17** |
| **3** | 418.2296 | 209.6184 | **E** | 1875.7936 | 938.4004 | 1859.7749 | 930.3911 | 1860.7827 | 930.8950 | **16** |
| **4** | 515.2824 | 258.1448 | **P** | 1732.7353 | 866.8713 | 1716.7166 | 858.8619 | 1717.7244 | 859.3659 | **15** |
| **5** | ***586.3195*** | 293.6634 | **A** | 1635.6826 | 818.3449 | 1619.6639 | 810.3356 | 1620.6717 | 810.8395 | **14** |
| **6** | ***729.3777*** | 365.1925 | **E** | 1564.6455 | 782.8264 | 1548.6267 | 774.8170 | 1549.6346 | 775.3209 | **13** |
| **7** | ***872.4360*** | 436.7216 | **E** | 1421.5872 | 711.2972 | 1405.5685 | 703.2879 | 1406.5763 | 703.7918 | **12** |
| **8** | ***1039.4343*** | 520.2208 | **S** | 1278.5290 | 639.7681 | 1262.5103 | 631.7588 | 1263.5181 | 632.2627 | **11** |
| **9** | ***1168.4769*** | 584.7421 | **D** | 1111.5306 | 556.2689 | 1095.5119 | 548.2596 | 1096.5197 | 548.7635 | **10** |
| **10** | ***1225.4984*** | 613.2528 | **G** | 982.4880 | 491.7477 | 966.4693 | 483.7383 | 967.4771 | 484.2422 | **9** |
| **11** | ***1354.5410*** | 677.7741 | **D** | 925.4666 | 463.2369 | 909.4478 | 455.2276 | 910.4557 | 455.7315 | **8** |
| **12** | ***1467.6251*** | 734.3162 | **L** | 796.4240 | 398.7156 | 780.4052 | 390.7063 | 781.4131 | 391.2102 | **7** |
| **13** | ***1524.6465*** | 762.8269 | **G** | 683.3399 | 342.1736 | 667.3212 | 334.1642 | 668.3290 | 334.6681 | **6** |
| **14** | ***1671.7149*** | 836.3611 | **F** | 626.3184 | 313.6629 | 610.2997 | 305.6535 | 611.3075 | 306.1574 | **5** |
| **15** | ***1728.7364*** | 864.8718 | **G** | 479.2500 | 240.1287 | 463.2313 | 232.1193 | 464.2391 | 232.6232 | **4** |
| **16** | ***1841.8205*** | 921.4139 | **L** | 422.2286 | 211.6179 | 406.2098 | 203.6086 | 407.2177 | 204.1125 | **3** |
| **17** | ***1988.8889*** | 994.9481 | **F** | 309.1445 | 155.0759 | 293.1258 | 147.0665 | 294.1336 | 147.5704 | **2** |
| **18** |  |  | **D** | 162.0761 | 81.5417 | 146.0574 | 73.5323 | 147.0652 | 74.0362 | **1** |

**CID** MS/MS Fragmentation of **SESLAK**
Found in **RS61_ARATH**, 40S ribosomal protein S6-1 OS=Arabidopsis thaliana GN=RPS6A PE=1 SV=2

Match to Query 6: 741.245448 from(371.630000,2+)


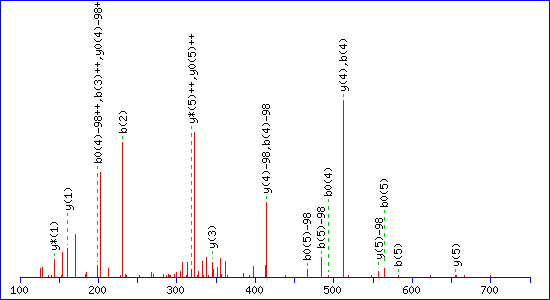


**Monoisotopic mass of neutral peptide Mr(calc):** 741.3309

**Fixed modifications:** Methyl (C-term),Methyl (DE)

**Variable modifications:**

**S3 :** Phospho (ST), with neutral losses 0.0000(shown in table), 97.9769

**Ions Score:** 21 **Expect:** 0.19

**Matches :** 21/68 fragment ions using 34 most intense peaks

| **#** | **b** | **b^++^** | **b^0^** | **b^0++^** | **Seq.** | **y** | **y^++^** | **y*** | **y*^++^** | **y^0^** | **y^0++^** | **#** |
| --- | --- | --- | --- | --- | --- | --- | --- | --- | --- | --- | --- | --- |
| **1** | 88.0393 | 44.5233 | 70.0287 | 35.5180 | **S** |  |  |  |  |  |  | **6** |
| **2** | ***231.0975*** | 116.0524 | 213.0870 | 107.0471 | **E** | ***655.3062*** | 328.1568 | 638.2797 | 319.6435 | 637.2957 | 319.1515 | **5** |
| **3** | 398.0959 | 199.5516 | 380.0853 | 190.5463 | **S** | ***512.2480*** | 256.6276 | 495.2214 | 248.1144 | 494.2374 | 247.6224 | **4** |
| **4** | ***511.1800*** | 256.0936 | 493.1694 | 247.0883 | **L** | ***345.2496*** | 173.1285 | 328.2231 | 164.6152 |  |  | **3** |
| **5** | ***582.2171*** | 291.6122 | 564.2065 | 282.6069 | **A** | 232.1656 | 116.5864 | 215.1390 | 108.0731 |  |  | **2** |
| **6** |  |  |  |  | **K** | ***161.1285*** | 81.0679 | 144.1019 | 72.5546 |  |  | **1** |

**ETD** MS/MS Fragmentation of **SESLAK**
Found in **RS61_ARATH**, 40S ribosomal protein S6-1 OS=Arabidopsis thaliana GN=RPS6A PE=1 SV=2

Match to Query 2: 741.225448 from(371.620000,2+)


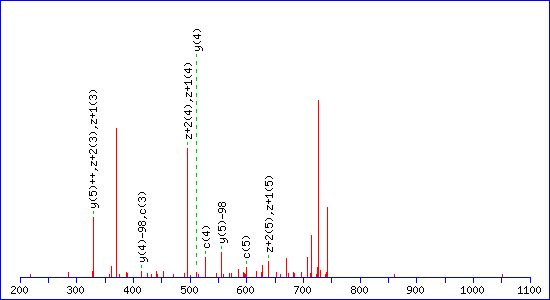


**Monoisotopic mass of neutral peptide Mr(calc):** 741.3309

**Fixed modifications:** Methyl (C-term),Methyl (DE)

**Variable modifications:**

**S3 :** Phospho (ST), with neutral losses 0.0000(shown in table), 97.9769

**Ions Score:** 22 **Expect:** 0.17

**Matches :** 9/58 fragment ions using 10 most intense peaks

| **#** | **c** | **c^++^** | **Seq.** | **y** | **y^++^** | **z+1** | **z+1^++^** | **z+2** | **z+2^++^** | **#** |
| --- | --- | --- | --- | --- | --- | --- | --- | --- | --- | --- |
| **1** | 105.0659 | 53.0366 | **S** |  |  |  |  |  |  | **6** |
| **2** | 248.1241 | 124.5657 | **E** | 655.3062 | 328.1568 | ***639.2875*** | 320.1474 | ***640.2953*** | 320.6513 | **5** |
| **3** | 415.1225 | 208.0649 | **S** | 512.2480 | 256.6276 | ***496.2293*** | 248.6183 | ***497.2371*** | 249.1222 | **4** |
| **4** | ***528.2065*** | 264.6069 | **L** | 345.2496 | 173.1285 | ***329.2309*** | 165.1191 | ***330.2387*** | 165.6230 | **3** |
| **5** | ***599.2436*** | 300.1255 | **A** | 232.1656 | 116.5864 | 216.1468 | 108.5771 | 217.1547 | 109.0810 | **2** |
| **6** |  |  | **K** | 161.1285 | 81.0679 | 145.1097 | 73.0585 | 146.1176 | 73.5624 | **1** |

**CID** MS/MS Fragmentation of **SESLAK**
Found in **RS61_ARATH**, 40S ribosomal protein S6-1 OS=Arabidopsis thaliana GN=RPS6A PE=1 SV=2

Match to Query 9: 741.282724 from(742.290000,1+)


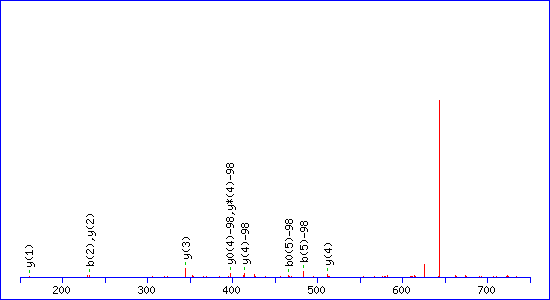


**Monoisotopic mass of neutral peptide Mr(calc):** 741.3309

**Fixed modifications:** Methyl (C-term),Methyl (DE)

**Variable modifications:**

**S3 :** Phospho (ST), with neutral losses 97.9769(shown in table), 0.0000

**Ions Score:** 24 **Expect:** 0.088

**Matches :** 10/34 fragment ions using 18 most intense peaks

| **#** | **b** | **b^0^** | **Seq.** | **y** | **y*** | **y^0^** | **#** |
| --- | --- | --- | --- | --- | --- | --- | --- |
| **1** | 88.0393 | 70.0287 | **S** |  |  |  | **6** |
| **2** | **231.0975** | 213.0870 | **E** | 557.3293 | 540.3028 | 539.3188 | **5** |
| **3** | 300.1190 | 282.1084 | **S** | ***414.2711*** | 397.2445 | 396.2605 | **4** |
| **4** | 413.2031 | 395.1925 | **L** | ***345.2496*** | 328.2231 |  | **3** |
| **5** | **484.2402** | 466.2296 | **A** | ***232.1656*** | 215.1390 |  | **2** |
| **6** |  |  | **K** | ***161.1285*** | 144.1019 |  | **1** |

**CID** MS/MS Fragmentation of **RSESLAK**
Found in **RS61_ARATH**, 40S ribosomal protein S6-1 OS=Arabidopsis thaliana GN=RPS6A PE=1 SV=2

Match to Query 15: 897.345448 from(449.680000,2+)


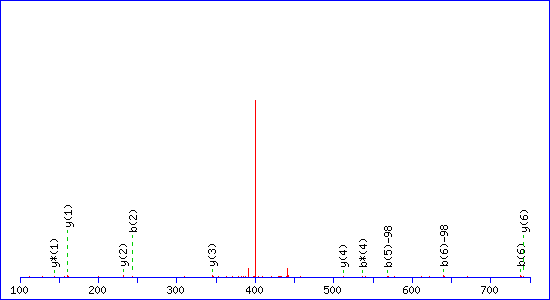


**Monoisotopic mass of neutral peptide Mr(calc):** 897.4321

**Fixed modifications:** Methyl (C-term),Methyl (DE)

**Variable modifications:**

**S4 :** Phospho (ST), with neutral losses 0.0000(shown in table), 97.9769

**Ions Score:** 36 **Expect:** 0.0051

**Matches :** 11/100 fragment ions using 21 most intense peaks

| **#** | **b** | **b^++^** | **b*** | **b*^++^** | **b^0^** | **b^0++^** | **Seq.** | **y** | **y^++^** | **y*** | **y*^++^** | **y^0^** | **y^0++^** | **#** |
| --- | --- | --- | --- | --- | --- | --- | --- | --- | --- | --- | --- | --- | --- | --- |
| **1** | 157.1084 | 79.0578 | 140.0818 | 70.5446 |  |  | **R** |  |  |  |  |  |  | **7** |
| **2** | **244.1404** | 122.5738 | 227.1139 | 114.0606 | 226.1298 | 113.5686 | **S** | ***742.3383*** | 371.6728 | 725.3117 | 363.1595 | 724.3277 | 362.6675 | **6** |
| **3** | 387.1987 | 194.1030 | 370.1721 | 185.5897 | 369.1881 | 185.0977 | **E** | 655.3062 | 328.1568 | 638.2797 | 319.6435 | 637.2957 | 319.1515 | **5** |
| **4** | 554.1970 | 277.6021 | 537.1705 | 269.0889 | 536.1865 | 268.5969 | **S** | ***512.2480*** | 256.6276 | 495.2214 | 248.1144 | 494.2374 | 247.6224 | **4** |
| **5** | 667.2811 | 334.1442 | 650.2545 | 325.6309 | 649.2705 | 325.1389 | **L** | ***345.2496*** | 173.1285 | 328.2231 | 164.6152 |  |  | **3** |
| **6** | **738.3182** | 369.6627 | 721.2916 | 361.1495 | 720.3076 | 360.6575 | **A** | ***232.1656*** | 116.5864 | 215.1390 | 108.0731 |  |  | **2** |
| **7** |  |  |  |  |  |  | **K** | ***161.1285*** | 81.0679 | 144.1019 | 72.5546 |  |  | **1** |

**ETD** MS/MS Fragmentation of **RSESLAK**
Found in **O48549|RS61_ARATH**, 40S ribosomal protein S6-1 - Arabidopsis thaliana (Mouse-ear cress)

Match to Query 8: 897.325448 from(449.670000,2+)


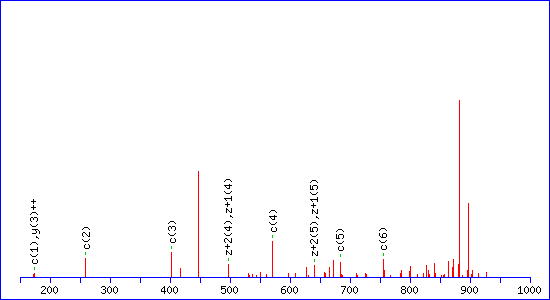


**Monoisotopic mass of neutral peptide Mr(calc):** 897.4321

**Fixed modifications:** Methyl (C-term),Methyl (DE)

**Variable modifications:**

**S4 :** Phospho (ST), with neutral losses 0.0000(shown in table), 97.9769

**Ions Score:** 44 **Expect:** 0.00071

**Matches :** 11/72 fragment ions using 14 most intense peaks

| **#** | **c** | **c++** | **Seq.** | **y** | **y++** | **z+1** | **z+1++** | **z+2** | **z+2++** | **#** |
| --- | --- | --- | --- | --- | --- | --- | --- | --- | --- | --- |
| **1** | ***174.1349*** | 87.5711 | **R** |  |  |  |  |  |  | **7** |
| **2** | ***261.1670*** | 131.0871 | **S** | 742.3383 | 371.6728 | 726.3195 | 363.6634 | 727.3274 | 364.1673 | **6** |
| **3** | ***404.2252*** | 202.6162 | **E** | 655.3062 | 328.1568 | **639.2875** | 320.1474 | **640.2953** | 320.6513 | **5** |
| **4** | ***571.2236*** | 286.1154 | **S** | 512.2480 | 256.6276 | **496.2293** | 248.6183 | **497.2371** | 249.1222 | **4** |
| **5** | ***684.3076*** | 342.6575 | **L** | 345.2496 | 173.1285 | 329.2309 | 165.1191 | 330.2387 | 165.6230 | **3** |
| **6** | ***755.3447*** | 378.1760 | **A** | 232.1656 | 116.5864 | 216.1468 | 108.5771 | 217.1547 | 109.0810 | **2** |
| **7** |  |  | **K** | 161.1285 | 81.0679 | 145.1097 | 73.0585 | 146.1176 | 73.5624 | **1** |

**CID** MS/MS Fragmentation of **SRLSSAPAKPVAA**
Found in **P51430|RS62_ARATH**, 40S ribosomal protein S6-2 - Arabidopsis thaliana (Mouse-ear cress)

Match to Query 5: 1347.525448 from(674.770000,2+)


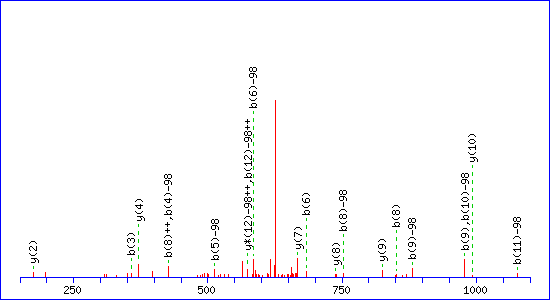


**Monoisotopic mass of neutral peptide Mr(calc):** 1347.6911

**Fixed modifications:** Methyl (C-term),Methyl (DE)

**Variable modifications:**

**S4 :** Phospho (ST), with neutral losses 97.9769(shown in table), 0.0000

**Ions Score:** 65 **Expect:** 0.0001

**Matches :** 20/190 fragment ions using 23 most intense peaks

| **#** | **b** | **b^++^** | **b*** | **b*^++^** | **b^0^** | **b^0++^** | **Seq.** | **y** | **y^++^** | **y*** | **y*^++^** | **y^0^** | **y^0++^** | **#** |
| --- | --- | --- | --- | --- | --- | --- | --- | --- | --- | --- | --- | --- | --- | --- |
| **1** | 88.0393 | 44.5233 |  |  | 70.0287 | 35.5180 | **S** |  |  |  |  |  |  | **13** |
| **2** | 244.1404 | 122.5738 | 227.1139 | 114.0606 | 226.1298 | 113.5686 | **R** | 1163.6895 | 582.3484 | 1146.6630 | 573.8351 | 1145.6790 | 573.3431 | **12** |
| **3** | ***357.2245*** | 179.1159 | 340.1979 | 170.6026 | 339.2139 | 170.1106 | **L** | 1007.5884 | 504.2978 | 990.5619 | 495.7846 | 989.5778 | 495.2926 | **11** |
| **4** | ***426.2459*** | 213.6266 | 409.2194 | 205.1133 | 408.2354 | 204.6213 | **S** | 894.5043 | 447.7558 | 877.4778 | 439.2425 | 876.4938 | 438.7505 | **10** |
| **5** | ***513.2780*** | 257.1426 | 496.2514 | 248.6293 | 495.2674 | 248.1373 | **S** | **825.4829** | 413.2451 | 808.4563 | 404.7318 | 807.4723 | 404.2398 | **9** |
| **6** | ***584.3151*** | 292.6612 | 567.2885 | 284.1479 | 566.3045 | 283.6559 | **A** | **738.4509** | 369.7291 | 721.4243 | 361.2158 |  |  | **8** |
| **7** | 681.3678 | 341.1876 | 664.3413 | 332.6743 | 663.3573 | 332.1823 | **P** | **667.4137** | 334.2105 | 650.3872 | 325.6972 |  |  | **7** |
| **8** | ***752.4050*** | 376.7061 | 735.3784 | 368.1928 | 734.3944 | 367.7008 | **A** | 570.3610 | 285.6841 | 553.3344 | 277.1709 |  |  | **6** |
| **9** | ***880.4999*** | 440.7536 | 863.4734 | 432.2403 | 862.4894 | 431.7483 | **K** | 499.3239 | 250.1656 | 482.2973 | 241.6523 |  |  | **5** |
| **10** | ***977.5527*** | 489.2800 | 960.5261 | 480.7667 | 959.5421 | 480.2747 | **P** | **371.2289** | 186.1181 |  |  |  |  | **4** |
| **11** | ***1076.6211*** | 538.8142 | 1059.5946 | 530.3009 | 1058.6105 | 529.8089 | **V** | 274.1761 | 137.5917 |  |  |  |  | **3** |
| **12** | 1147.6582 | 574.3327 | 1130.6317 | 565.8195 | 1129.6477 | 565.3275 | **A** | **175.1077** | 88.0575 |  |  |  |  | **2** |
| **13** |  |  |  |  |  |  | **A** | 104.0706 | 52.5389 |  |  |  |  | **1** |

**ETD** MS/MS Fragmentation of **SRLSSAPAKPVAA**
Found in **P51430|RS62_ARATH**, 40S ribosomal protein S6-2 - Arabidopsis thaliana (Mouse-ear cress)

Match to Query 4: 1347.545448 from(674.780000,2+)


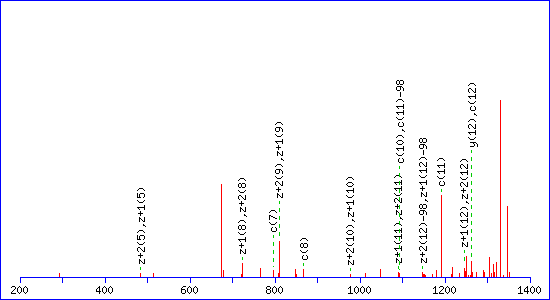


**Monoisotopic mass of neutral peptide Mr(calc):** 1347.6911

**Fixed modifications:** Methyl (C-term),Methyl (DE)

**Variable modifications:**

**S4 :** Phospho (ST), with neutral losses 0.0000(shown in table), 97.9769

**Ions Score:** 32 **Expect:** 0.23

**Matches :** 21/132 fragment ions using 29 most intense peaks

| **#** | **c** | **c^++^** | **Seq.** | **y** | **y^++^** | **z+1** | **z+1^++^** | **z+2** | **z+2^++^** | **#** |
| --- | --- | --- | --- | --- | --- | --- | --- | --- | --- | --- |
| **1** | 105.0659 | 53.0366 | **S** |  |  |  |  |  |  | **13** |
| **2** | 261.1670 | 131.0871 | **R** | 1261.6664 | 631.3368 | ***1245.6477*** | 623.3275 | ***1246.6555*** | 623.8314 | **12** |
| **3** | 374.2510 | 187.6292 | **L** | 1105.5653 | 553.2863 | ***1089.5466*** | 545.2769 | ***1090.5544*** | 545.7808 | **11** |
| **4** | 541.2494 | 271.1283 | **S** | 992.4812 | 496.7443 | ***976.4625*** | 488.7349 | ***977.4703*** | 489.2388 | **10** |
| **5** | 628.2814 | 314.6443 | **S** | 825.4829 | 413.2451 | ***809.4642*** | 405.2357 | ***810.4720*** | 405.7396 | **9** |
| **6** | 699.3185 | 350.1629 | **A** | 738.4509 | 369.7291 | ***722.4321*** | 361.7197 | ***723.4400*** | 362.2236 | **8** |
| **7** | ***796.3713*** | 398.6893 | **P** | 667.4137 | 334.2105 | 651.3950 | 326.2011 | 652.4028 | 326.7051 | **7** |
| **8** | ***867.4084*** | 434.2078 | **A** | 570.3610 | 285.6841 | 554.3423 | 277.6748 | 555.3501 | 278.1787 | **6** |
| **9** | 995.5034 | 498.2553 | **K** | 499.3239 | 250.1656 | ***483.3051*** | 242.1562 | ***484.3130*** | 242.6601 | **5** |
| **10** | ***1092.5561*** | 546.7817 | **P** | 371.2289 | 186.1181 | 355.2102 | 178.1087 | 356.2180 | 178.6126 | **4** |
| **11** | ***1191.6245*** | 596.3159 | **V** | 274.1761 | 137.5917 | 258.1574 | 129.5823 | 259.1652 | 130.0863 | **3** |
| **12** | ***1262.6617*** | 631.8345 | **A** | 175.1077 | 88.0575 | 159.0890 | 80.0481 | 160.0968 | 80.5520 | **2** |
| **13** |  |  | **A** | 104.0706 | 52.5389 | 88.0519 | 44.5296 | 89.0597 | 45.0335 | **1** |

**CID** MS/MS Fragmentation of **SRLSSAPAKPVAA**
Found in **RS62_ARATH**, 40S ribosomal protein S6-2 OS=Arabidopsis thaliana GN=RPS6B PE=1 SV=3

Match to Query 2: 1427.445448 from(714.730000,2+)


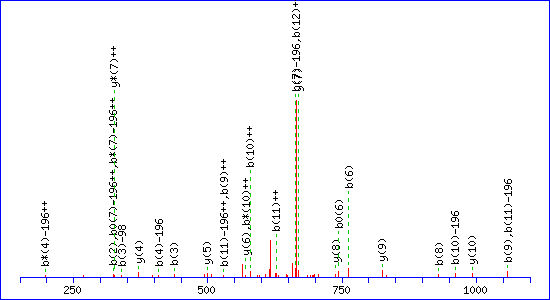


**Monoisotopic mass of neutral peptide Mr(calc):** 1427.6574

**Fixed modifications:** Methyl (C-term),Methyl (DE)

**Variable modifications:**

**S1 :** Phospho (ST), with neutral losses 0.0000(shown in table), 97.9769

**S4 :** Phospho (ST), with neutral losses 0.0000(shown in table), 97.9769

**Ions Score:** 42 **Expect:** 0.0021

**Matches :** 26/206 fragment ions using 47 most intense peaks

| **#** | **b** | **b^++^** | **b*** | **b*^++^** | **b^0^** | **b^0++^** | **Seq.** | **y** | **y^++^** | **y*** | **y*^++^** | **y^0^** | **y^0++^** | **#** |
| --- | --- | --- | --- | --- | --- | --- | --- | --- | --- | --- | --- | --- | --- | --- |
| **1** | 168.0056 | 84.5065 |  |  | 149.9951 | 75.5012 | **S** |  |  |  |  |  |  | **13** |
| **2** | **324.1067** | 162.5570 | 307.0802 | 154.0437 | 306.0962 | 153.5517 | **R** | 1261.6664 | 631.3368 | 1244.6399 | 622.8236 | 1243.6558 | 622.3316 | **12** |
| **3** | **437.1908** | 219.0990 | 420.1643 | 210.5858 | 419.1802 | 210.0938 | **L** | 1105.5653 | 553.2863 | 1088.5388 | 544.7730 | 1087.5547 | 544.2810 | **11** |
| **4** | 604.1892 | 302.5982 | 587.1626 | 294.0849 | 586.1786 | 293.5929 | **S** | ***992.4812*** | 496.7443 | 975.4547 | 488.2310 | 974.4707 | 487.7390 | **10** |
| **5** | 691.2212 | 346.1142 | 674.1946 | 337.6010 | 673.2106 | 337.1090 | **S** | ***825.4829*** | 413.2451 | 808.4563 | 404.7318 | 807.4723 | 404.2398 | **9** |
| **6** | **762.2583** | 381.6328 | 745.2318 | 373.1195 | 744.2477 | 372.6275 | **A** | ***738.4509*** | 369.7291 | 721.4243 | 361.2158 |  |  | **8** |
| **7** | 859.3111 | 430.1592 | 842.2845 | 421.6459 | 841.3005 | 421.1539 | **P** | ***667.4137*** | 334.2105 | 650.3872 | 325.6972 |  |  | **7** |
| **8** | **930.3482** | 465.6777 | 913.3216 | 457.1645 | 912.3376 | 456.6724 | **A** | ***570.3610*** | 285.6841 | 553.3344 | 277.1709 |  |  | **6** |
| **9** | **1058.4432** | ***529.7252*** | 1041.4166 | 521.2119 | 1040.4326 | 520.7199 | **K** | ***499.3239*** | 250.1656 | 482.2973 | 241.6523 |  |  | **5** |
| **10** | 1155.4959 | ***578.2516*** | 1138.4694 | 569.7383 | 1137.4854 | 569.2463 | **P** | ***371.2289*** | 186.1181 |  |  |  |  | **4** |
| **11** | 1254.5643 | ***627.7858*** | 1237.5378 | 619.2725 | 1236.5538 | 618.7805 | **V** | 274.1761 | 137.5917 |  |  |  |  | **3** |
| **12** | 1325.6014 | ***663.3044*** | 1308.5749 | 654.7911 | 1307.5909 | 654.2991 | **A** | 175.1077 | 88.0575 |  |  |  |  | **2** |
| **13** |  |  |  |  |  |  | **A** | 104.0706 | 52.5389 |  |  |  |  | **1** |

**ETD** MS/MS Fragmentation of **SRLSSAPAKPVAA**
Found in **RS62_ARATH**, 40S ribosomal protein S6-2 OS=Arabidopsis thaliana GN=RPS6B PE=1 SV=3

Match to Query 2: 1427.445448 from(714.730000,2+)


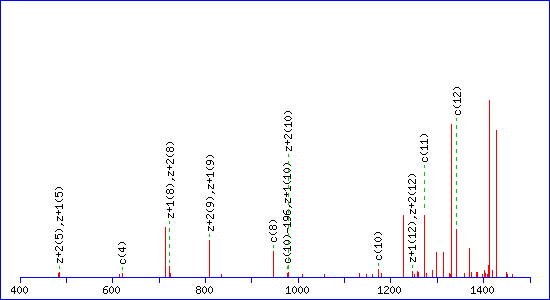


**Monoisotopic mass of neutral peptide Mr(calc):** 1427.6574

**Fixed modifications:** Methyl (C-term),Methyl (DE)

**Variable modifications:**

**S1 :** Phospho (ST), with neutral losses 0.0000(shown in table), 97.9769

**S4 :** Phospho (ST), with neutral losses 0.0000(shown in table), 97.9769

**Ions Score:** 35 **Expect:** 0.011

**Matches :** 16/138 fragment ions using 26 most intense peaks

| **#** | **c** | **c^++^** | **Seq.** | **y** | **y^++^** | **z+1** | **z+1^++^** | **z+2** | **z+2^++^** | **#** |
| --- | --- | --- | --- | --- | --- | --- | --- | --- | --- | --- |
| **1** | 185.0322 | 93.0197 | **S** |  |  |  |  |  |  | **13** |
| **2** | 341.1333 | 171.0703 | **R** | 1261.6664 | 631.3368 | ***1245.6477*** | 623.3275 | **1246.6555** | 623.8314 | **12** |
| **3** | 454.2174 | 227.6123 | **L** | 1105.5653 | 553.2863 | 1089.5466 | 545.2769 | 1090.5544 | 545.7808 | **11** |
| **4** | ***621.2157*** | 311.1115 | **S** | 992.4812 | 496.7443 | ***976.4625*** | 488.7349 | **977.4703** | 489.2388 | **10** |
| **5** | 708.2477 | 354.6275 | **S** | 825.4829 | 413.2451 | ***809.4642*** | 405.2357 | **810.4720** | 405.7396 | **9** |
| **6** | 779.2849 | 390.1461 | **A** | 738.4509 | 369.7291 | ***722.4321*** | 361.7197 | **723.4400** | 362.2236 | **8** |
| **7** | 876.3376 | 438.6725 | **P** | 667.4137 | 334.2105 | 651.3950 | 326.2011 | 652.4028 | 326.7051 | **7** |
| **8** | ***947.3747*** | 474.1910 | **A** | 570.3610 | 285.6841 | 554.3423 | 277.6748 | 555.3501 | 278.1787 | **6** |
| **9** | 1075.4697 | 538.2385 | **K** | 499.3239 | 250.1656 | ***483.3051*** | 242.1562 | **484.3130** | 242.6601 | **5** |
| **10** | ***1172.5225*** | 586.7649 | **P** | 371.2289 | 186.1181 | 355.2102 | 178.1087 | 356.2180 | 178.6126 | **4** |
| **11** | ***1271.5909*** | 636.2991 | **V** | 274.1761 | 137.5917 | 258.1574 | 129.5823 | 259.1652 | 130.0863 | **3** |
| **12** | ***1342.6280*** | 671.8176 | **A** | 175.1077 | 88.0575 | 159.0890 | 80.0481 | 160.0968 | 80.5520 | **2** |
| **13** |  |  | **A** | 104.0706 | 52.5389 | 88.0519 | 44.5296 | 89.0597 | 45.0335 | **1** |

**CID** MS/MS Fragmentation of **SRLSSAAAK**
Found in **RS61_ARATH**, 40S ribosomal protein S6-1 OS=Arabidopsis thaliana GN=RPS6A PE=1 SV=2

Match to Query 180: 983.345448 from(492.680000,2+)


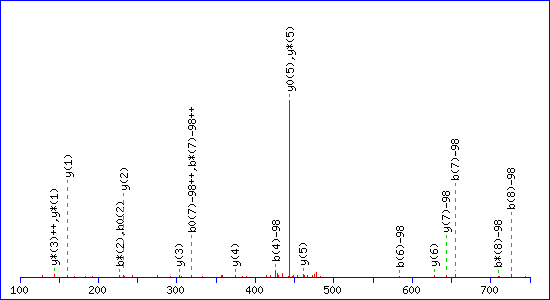


**Monoisotopic mass of neutral peptide Mr(calc):** 983.4801

**Fixed modifications:** Methyl (C-term),Methyl (DE)

**Variable modifications:**

**S4 :** Phospho (ST), with neutral losses 97.9769(shown in table), 0.0000

**Ions Score:** 50 **Expect:** 0.0005

**Matches :** 18/134 fragment ions using 23 most intense peaks

| **#** | **b** | **b^++^** | **b*** | **b*^++^** | **b^0^** | **b^0++^** | **Seq.** | **y** | **y^++^** | **y*** | **y*^++^** | **y^0^** | **y^0++^** | **#** |
| --- | --- | --- | --- | --- | --- | --- | --- | --- | --- | --- | --- | --- | --- | --- |
| **1** | 88.0393 | 44.5233 |  |  | 70.0287 | 35.5180 | **S** |  |  |  |  |  |  | **9** |
| **2** | 244.1404 | 122.5738 | 227.1139 | 114.0606 | 226.1298 | 113.5686 | **R** | 799.4785 | 400.2429 | 782.4519 | 391.7296 | 781.4679 | 391.2376 | **8** |
| **3** | 357.2245 | 179.1159 | 340.1979 | 170.6026 | 339.2139 | 170.1106 | **L** | ***643.3774*** | 322.1923 | 626.3508 | 313.6790 | 625.3668 | 313.1870 | **7** |
| **4** | **426.2459** | 213.6266 | 409.2194 | 205.1133 | 408.2354 | 204.6213 | **S** | 530.2933 | 265.6503 | 513.2667 | 257.1370 | 512.2827 | 256.6450 | **6** |
| **5** | 513.2780 | 257.1426 | 496.2514 | 248.6293 | 495.2674 | 248.1373 | **S** | ***461.2718*** | 231.1395 | 444.2453 | 222.6263 | 443.2613 | 222.1343 | **5** |
| **6** | **584.3151** | 292.6612 | 567.2885 | 284.1479 | 566.3045 | 283.6559 | **A** | ***374.2398*** | 187.6235 | 357.2132 | 179.1103 |  |  | **4** |
| **7** | **655.3522** | 328.1797 | 638.3256 | 319.6665 | 637.3416 | 319.1745 | **A** | ***303.2027*** | 152.1050 | 286.1761 | 143.5917 |  |  | **3** |
| **8** | **726.3893** | 363.6983 | 709.3628 | 355.1850 | 708.3787 | 354.6930 | **A** | ***232.1656*** | 116.5864 | 215.1390 | 108.0731 |  |  | **2** |
| **9** |  |  |  |  |  |  | **K** | ***161.1285*** | 81.0679 | 144.1019 | 72.5546 |  |  | **1** |

**ETD** MS/MS Fragmentation of **SRLSSAAAK**
Found in **RS61_ARATH**, 40S ribosomal protein S6-1 OS=Arabidopsis thaliana GN=RPS6A PE=1 SV=2

Match to Query 90: 983.345448 from(492.680000,2+)


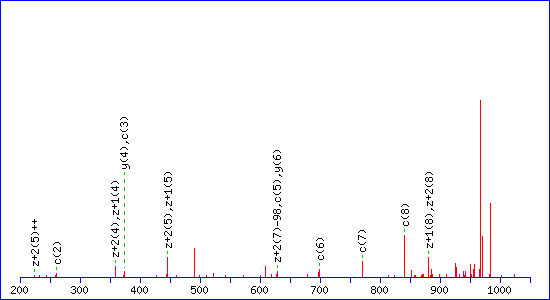


**Monoisotopic mass of neutral peptide Mr(calc):** 983.4801

**Fixed modifications:** Methyl (C-term),Methyl (DE)

**Variable modifications:**

**S4 :** Phospho (ST), with neutral losses 0.0000(shown in table), 97.9769

**Ions Score:** 46 **Expect:** 0.0007

**Matches :** 16/92 fragment ions using 16 most intense peaks

| **#** | **c** | **c^++^** | **Seq.** | **y** | **y^++^** | **z+1** | **z+1^++^** | **z+2** | **z+2^++^** | **#** |
| --- | --- | --- | --- | --- | --- | --- | --- | --- | --- | --- |
| **1** | 105.0659 | 53.0366 | **S** |  |  |  |  |  |  | **9** |
| **2** | ***261.1670*** | 131.0871 | **R** | 897.4554 | 449.2313 | **881.4366** | 441.2220 | **882.4445** | 441.7259 | **8** |
| **3** | ***374.2510*** | 187.6292 | **L** | 741.3542 | 371.1808 | 725.3355 | 363.1714 | 726.3433 | 363.6753 | **7** |
| **4** | 541.2494 | 271.1283 | **S** | 628.2702 | 314.6387 | 612.2515 | 306.6294 | 613.2593 | 307.1333 | **6** |
| **5** | ***628.2814*** | 314.6443 | **S** | 461.2718 | 231.1395 | **445.2531** | 223.1302 | **446.2609** | 223.6341 | **5** |
| **6** | ***699.3185*** | 350.1629 | **A** | 374.2398 | 187.6235 | **358.2211** | 179.6142 | **359.2289** | 180.1181 | **4** |
| **7** | ***770.3556*** | 385.6815 | **A** | 303.2027 | 152.1050 | 287.1840 | 144.0956 | 288.1918 | 144.5995 | **3** |
| **8** | ***841.3928*** | 421.2000 | **A** | 232.1656 | 116.5864 | 216.1468 | 108.5771 | 217.1547 | 109.0810 | **2** |
| **9** |  |  | **K** | 161.1285 | 81.0679 | 145.1097 | 73.0585 | 146.1176 | 73.5624 | **1** |

**CID** MS/MS Fragmentation of **SRLSSAAAKPSVTA**
Found in **RS61_ARATH**, 40S ribosomal protein S6-1 OS=Arabidopsis thaliana GN=RPS6A PE=1 SV=2

Match to Query 6: 1438.645448 from(720.330000,2+)


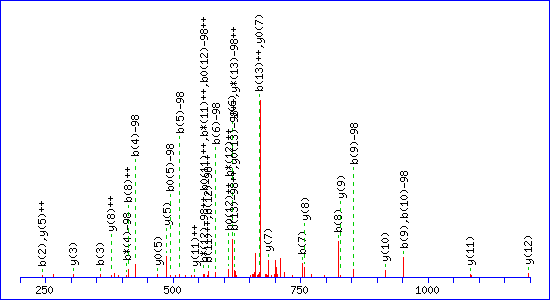


**Monoisotopic mass of neutral peptide Mr(calc):** 1438.7181

**Fixed modifications:** Methyl (C-term),Methyl (DE)

**Variable modifications:**

**S4 :** Phospho (ST), with neutral losses 0.0000(shown in table), 97.9769

**Ions Score:** 61 **Expect:** 4.1e-05

**Matches :** 26/220 fragment ions using 39 most intense peaks

| **#** | **b** | **b^++^** | **b*** | **b*^++^** | **b^0^** | **b^0++^** | **Seq.** | **y** | **y^++^** | **y*** | **y*^++^** | **y^0^** | **y^0++^** | **#** |
| --- | --- | --- | --- | --- | --- | --- | --- | --- | --- | --- | --- | --- | --- | --- |
| **1** | 88.0393 | 44.5233 |  |  | 70.0287 | 35.5180 | **S** |  |  |  |  |  |  | **14** |
| **2** | **244.1404** | 122.5738 | 227.1139 | 114.0606 | 226.1298 | 113.5686 | **R** | 1352.6934 | 676.8503 | 1335.6668 | 668.3370 | 1334.6828 | 667.8450 | **13** |
| **3** | **357.2245** | 179.1159 | 340.1979 | 170.6026 | 339.2139 | 170.1106 | **L** | ***1196.5922*** | 598.7998 | 1179.5657 | 590.2865 | 1178.5817 | 589.7945 | **12** |
| **4** | 524.2228 | 262.6151 | 507.1963 | 254.1018 | 506.2123 | 253.6098 | **S** | ***1083.5082*** | 542.2577 | 1066.4816 | 533.7445 | 1065.4976 | 533.2524 | **11** |
| **5** | 611.2549 | 306.1311 | 594.2283 | 297.6178 | 593.2443 | 297.1258 | **S** | ***916.5098*** | 458.7585 | 899.4833 | 450.2453 | 898.4993 | 449.7533 | **10** |
| **6** | 682.2920 | 341.6496 | 665.2654 | 333.1364 | 664.2814 | 332.6443 | **A** | ***829.4778*** | 415.2425 | 812.4512 | 406.7293 | 811.4672 | 406.2373 | **9** |
| **7** | **753.3291** | 377.1682 | 736.3025 | 368.6549 | 735.3185 | 368.1629 | **A** | ***758.4407*** | 379.7240 | 741.4141 | 371.2107 | 740.4301 | 370.7187 | **8** |
| **8** | **824.3662** | 412.6867 | 807.3397 | 404.1735 | 806.3556 | 403.6815 | **A** | ***687.4036*** | 344.2054 | 670.3770 | 335.6921 | 669.3930 | 335.2001 | **7** |
| **9** | **952.4612** | 476.7342 | 935.4346 | 468.2209 | 934.4506 | 467.7289 | **K** | ***616.3665*** | 308.6869 | 599.3399 | 300.1736 | 598.3559 | 299.6816 | **6** |
| **10** | 1049.5139 | 525.2606 | 1032.4874 | 516.7473 | 1031.5034 | 516.2553 | **P** | ***488.2715*** | 244.6394 |  |  | 470.2609 | 235.6341 | **5** |
| **11** | 1136.5460 | 568.7766 | 1119.5194 | 560.2633 | 1118.5354 | 559.7713 | **S** | 391.2187 | 196.1130 |  |  | 373.2082 | 187.1077 | **4** |
| **12** | 1235.6144 | 618.3108 | 1218.5878 | 609.7976 | 1217.6038 | 609.3055 | **V** | ***304.1867*** | 152.5970 |  |  | 286.1761 | 143.5917 | **3** |
| **13** | 1336.6621 | 668.8347 | 1319.6355 | 660.3214 | 1318.6515 | 659.8294 | **T** | 205.1183 | 103.0628 |  |  | 187.1077 | 94.0575 | **2** |
| **14** |  |  |  |  |  |  | **A** | 104.0706 | 52.5389 |  |  |  |  | **1** |

**ETD** MS/MS Fragmentation of **SRLSSAAAKPSVTA**
Found in **RS61_ARATH**, 40S ribosomal protein S6-1 OS=Arabidopsis thaliana GN=RPS6A PE=1 SV=2

Match to Query 3: 1438.565448 from(720.290000,2+)


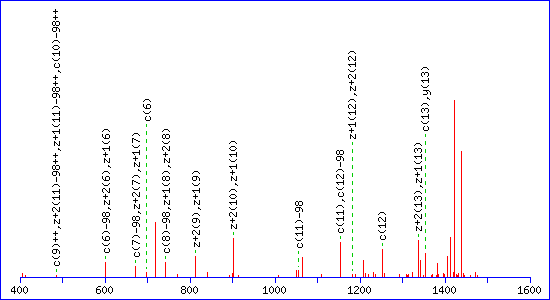


**Monoisotopic mass of neutral peptide Mr(calc):** 1438.7181

**Fixed modifications:** Methyl (C-term),Methyl (DE)

**Variable modifications:**

**S4 :** Phospho (ST), with neutral losses 0.0000(shown in table), 97.9769

**Ions Score:** 50 **Expect:** 0.00055

**Matches :** 28/142 fragment ions using 20 most intense peaks

| **#** | **c** | **c^++^** | **Seq.** | **y** | **y^++^** | **z+1** | **z+1^++^** | **z+2** | **z+2^++^** | **#** |
| --- | --- | --- | --- | --- | --- | --- | --- | --- | --- | --- |
| **1** | 105.0659 | 53.0366 | **S** |  |  |  |  |  |  | **14** |
| **2** | 261.1670 | 131.0871 | **R** | 1352.6934 | 676.8503 | ***1336.6746*** | 668.8410 | ***1337.6825*** | 669.3449 | **13** |
| **3** | 374.2510 | 187.6292 | **L** | 1196.5922 | 598.7998 | ***1180.5735*** | 590.7904 | ***1181.5813*** | 591.2943 | **12** |
| **4** | 541.2494 | 271.1283 | **S** | 1083.5082 | 542.2577 | 1067.4895 | 534.2484 | 1068.4973 | 534.7523 | **11** |
| **5** | 628.2814 | 314.6443 | **S** | 916.5098 | 458.7585 | ***900.4911*** | 450.7492 | ***901.4989*** | 451.2531 | **10** |
| **6** | **699.3185** | 350.1629 | **A** | 829.4778 | 415.2425 | ***813.4591*** | 407.2332 | ***814.4669*** | 407.7371 | **9** |
| **7** | 770.3556 | 385.6815 | **A** | 758.4407 | 379.7240 | ***742.4220*** | 371.7146 | ***743.4298*** | 372.2185 | **8** |
| **8** | 841.3928 | 421.2000 | **A** | 687.4036 | 344.2054 | ***671.3848*** | 336.1961 | ***672.3927*** | 336.7000 | **7** |
| **9** | 969.4877 | 485.2475 | **K** | 616.3665 | 308.6869 | ***600.3477*** | 300.6775 | ***601.3556*** | 301.1814 | **6** |
| **10** | 1066.5405 | 533.7739 | **P** | 488.2715 | 244.6394 | 472.2528 | 236.6300 | 473.2606 | 237.1339 | **5** |
| **11** | **1153.5725** | 577.2899 | **S** | 391.2187 | 196.1130 | 375.2000 | 188.1036 | 376.2078 | 188.6076 | **4** |
| **12** | **1252.6409** | 626.8241 | **V** | 304.1867 | 152.5970 | 288.1680 | 144.5876 | 289.1758 | 145.0915 | **3** |
| **13** | **1353.6886** | 677.3479 | **T** | 205.1183 | 103.0628 | 189.0996 | 95.0534 | 190.1074 | 95.5573 | **2** |
| **14** |  |  | **A** | 104.0706 | 52.5389 | 88.0519 | 44.5296 | 89.0597 | 45.0335 | **1** |

**CID** MS/MS Fragmentation of **SRLSSAAAKPSVTA**
Found in **RS61_ARATH**, 40S ribosomal protein S6-1 OS=Arabidopsis thaliana GN=RPS6A PE=1 SV=2

Match to Query 1: 1518.585448 from(760.300000,2+)

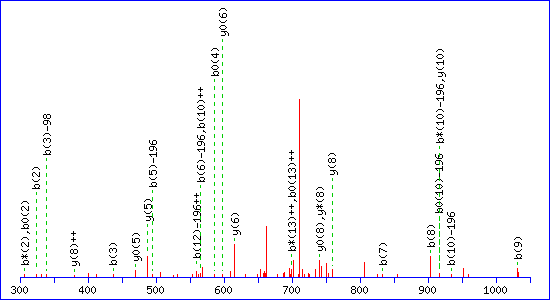


**Monoisotopic mass of neutral peptide Mr(calc):** 1518.6844

**Fixed modifications:** Methyl (C-term),Methyl (DE)

**Variable modifications:**

**S1 :** Phospho (ST), with neutral losses 0.0000(shown in table), 97.9769

**S4 :** Phospho (ST), with neutral losses 0.0000(shown in table), 97.9769

**Ions Score:** 21 **Expect:** 0.13

**Matches :** 25/236 fragment ions using 48 most intense peaks

| **#** | **b** | **b^++^** | **b*** | **b*^++^** | **b^0^** | **b^0++^** | **Seq.** | **y** | **y^++^** | **y*** | **y*^++^** | **y^0^** | **y^0++^** | **#** |
| --- | --- | --- | --- | --- | --- | --- | --- | --- | --- | --- | --- | --- | --- | --- |
| **1** | 168.0056 | 84.5065 |  |  | 149.9951 | 75.5012 | **S** |  |  |  |  |  |  | **14** |
| **2** | ***324.1067*** | 162.5570 | 307.0802 | 154.0437 | 306.0962 | 153.5517 | **R** | 1352.6934 | 676.8503 | 1335.6668 | 668.3370 | 1334.6828 | 667.8450 | **13** |
| **3** | ***437.1908*** | 219.0990 | 420.1643 | 210.5858 | 419.1802 | 210.0938 | **L** | 1196.5922 | 598.7998 | 1179.5657 | 590.2865 | 1178.5817 | 589.7945 | **12** |
| **4** | 604.1892 | 302.5982 | 587.1626 | 294.0849 | 586.1786 | 293.5929 | **S** | 1083.5082 | 542.2577 | 1066.4816 | 533.7445 | 1065.4976 | 533.2524 | **11** |
| **5** | 691.2212 | 346.1142 | 674.1946 | 337.6010 | 673.2106 | 337.1090 | **S** | ***916.5098*** | 458.7585 | 899.4833 | 450.2453 | 898.4993 | 449.7533 | **10** |
| **6** | 762.2583 | 381.6328 | 745.2318 | 373.1195 | 744.2477 | 372.6275 | **A** | 829.4778 | 415.2425 | 812.4512 | 406.7293 | 811.4672 | 406.2373 | **9** |
| **7** | ***833.2954*** | 417.1514 | 816.2689 | 408.6381 | 815.2849 | 408.1461 | **A** | ***758.4407*** | 379.7240 | 741.4141 | 371.2107 | 740.4301 | 370.7187 | **8** |
| **8** | ***904.3325*** | 452.6699 | 887.3060 | 444.1566 | 886.3220 | 443.6646 | **A** | 687.4036 | 344.2054 | 670.3770 | 335.6921 | 669.3930 | 335.2001 | **7** |
| **9** | ***1032.4275*** | 516.7174 | 1015.4010 | 508.2041 | 1014.4169 | 507.7121 | **K** | ***616.3665*** | 308.6869 | 599.3399 | 300.1736 | 598.3559 | 299.6816 | **6** |
| **10** | 1129.4803 | 565.2438 | 1112.4537 | 556.7305 | 1111.4697 | 556.2385 | **P** | ***488.2715*** | 244.6394 |  |  | 470.2609 | 235.6341 | **5** |
| **11** | 1216.5123 | 608.7598 | 1199.4857 | 600.2465 | 1198.5017 | 599.7545 | **S** | 391.2187 | 196.1130 |  |  | 373.2082 | 187.1077 | **4** |
| **12** | 1315.5807 | 658.2940 | 1298.5542 | 649.7807 | 1297.5701 | 649.2887 | **V** | 304.1867 | 152.5970 |  |  | 286.1761 | 143.5917 | **3** |
| **13** | 1416.6284 | 708.8178 | 1399.6018 | 700.3046 | 1398.6178 | 699.8125 | **T** | 205.1183 | 103.0628 |  |  | 187.1077 | 94.0575 | **2** |
| **14** |  |  |  |  |  |  | **A** | 104.0706 | 52.5389 |  |  |  |  | **1** |

**ETD** MS/MS Fragmentation of **SRLSSAAAKPSVTA**
Found in **RS61_ARATH**, 40S ribosomal protein S6-1 OS=Arabidopsis thaliana GN=RPS6A PE=1 SV=2

Match to Query 1: 1518.585448 from(760.300000,2+)


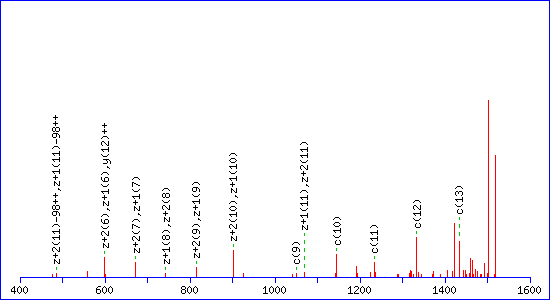


**Monoisotopic mass of neutral peptide Mr(calc):** 1518.6844

**Fixed modifications:** Methyl (C-term),Methyl (DE)

**Variable modifications:**

**S1 :** Phospho (ST), with neutral losses 0.0000(shown in table), 97.9769

**S4 :** Phospho (ST), with neutral losses 0.0000(shown in table), 97.9769

**Ions Score:** 39 **Expect:** 0.0017

**Matches :** 20/148 fragment ions using 21 most intense peaks

| **#** | **c** | **c++** | **Seq.** | **y** | **y++** | **z+1** | **z+1++** | **z+2** | **z+2++** | **#** |
| --- | --- | --- | --- | --- | --- | --- | --- | --- | --- | --- |
| **1** | 185.0322 | 93.0197 | **S** |  |  |  |  |  |  | **14** |
| **2** | 341.1333 | 171.0703 | **R** | 1352.6934 | 676.8503 | 1336.6746 | 668.8410 | 1337.6825 | 669.3449 | **13** |
| **3** | 454.2174 | 227.6123 | **L** | 1196.5922 | 598.7998 | 1180.5735 | 590.7904 | 1181.5813 | 591.2943 | **12** |
| **4** | 621.2157 | 311.1115 | **S** | 1083.5082 | 542.2577 | ***1067.4895*** | 534.2484 | ***1068.4973*** | 534.7523 | **11** |
| **5** | 708.2477 | 354.6275 | **S** | 916.5098 | 458.7585 | ***900.4911*** | 450.7492 | ***901.4989*** | 451.2531 | **10** |
| **6** | 779.2849 | 390.1461 | **A** | 829.4778 | 415.2425 | ***813.4591*** | 407.2332 | ***814.4669*** | 407.7371 | **9** |
| **7** | 850.3220 | 425.6646 | **A** | 758.4407 | 379.7240 | ***742.4220*** | 371.7146 | ***743.4298*** | 372.2185 | **8** |
| **8** | 921.3591 | 461.1832 | **A** | 687.4036 | 344.2054 | ***671.3848*** | 336.1961 | ***672.3927*** | 336.7000 | **7** |
| **9** | ***1049.4541*** | 525.2307 | **K** | 616.3665 | 308.6869 | ***600.3477*** | 300.6775 | ***601.3556*** | 301.1814 | **6** |
| **10** | ***1146.5068*** | 573.7570 | **P** | 488.2715 | 244.6394 | 472.2528 | 236.6300 | 473.2606 | 237.1339 | **5** |
| **11** | ***1233.5388*** | 617.2731 | **S** | 391.2187 | 196.1130 | 375.2000 | 188.1036 | 376.2078 | 188.6076 | **4** |
| **12** | ***1332.6073*** | 666.8073 | **V** | 304.1867 | 152.5970 | 288.1680 | 144.5876 | 289.1758 | 145.0915 | **3** |
| **13** | ***1433.6549*** | 717.3311 | **T** | 205.1183 | 103.0628 | 189.0996 | 95.0534 | 190.1074 | 95.5573 | **2** |
| **14** |  |  | **A** | 104.0706 | 52.5389 | 88.0519 | 44.5296 | 89.0597 | 45.0335 | **1** |

**CID** MS/MS Fragmentation of **VEEKEESDEEDYG**
Found in **RLA02_ARATH**, 60S acidic ribosomal protein P0-2 OS=Arabidopsis thaliana GN=RPP0B PE=1 SV=2

Match to Query 1: 1762.525448 from(882.270000,2+)


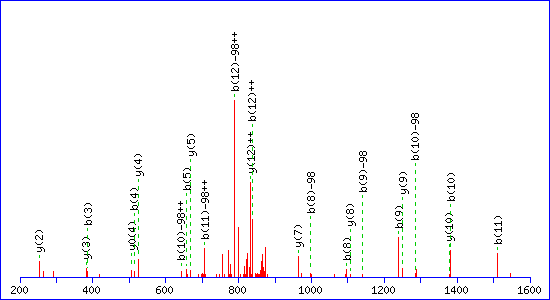


**Monoisotopic mass of neutral peptide Mr(calc):** 1762.7074

**Fixed modifications:** Methyl (C-term),Methyl (DE)

**Variable modifications:**

**S7 :** Phospho (ST), with neutral losses 0.0000(shown in table), 97.9769

**Ions Score:** 54 **Expect:** 0.0014

**Matches :** 24/180 fragment ions using 34 most intense peaks

| **#** | **b** | **b++** | **b*** | **b*++** | **b0** | **b0++** | **Seq.** | **y** | **y++** | **y*** | **y*++** | **y0** | **y0++** | **#** |
| --- | --- | --- | --- | --- | --- | --- | --- | --- | --- | --- | --- | --- | --- | --- |
| **1** | 100.0757 | 50.5415 |  |  |  |  | **V** |  |  |  |  |  |  | **13** |
| **2** | 243.1339 | 122.0706 |  |  | 225.1234 | 113.0653 | **E** | 1664.6463 | 832.8268 | 1647.6197 | 824.3135 | 1646.6357 | 823.8215 | **12** |
| **3** | **386.1922** | 193.5997 |  |  | 368.1816 | 184.5944 | **E** | 1521.5880 | 761.2976 | 1504.5615 | 752.7844 | 1503.5774 | 752.2924 | **11** |
| **4** | **514.2871** | 257.6472 | 497.2606 | 249.1339 | 496.2766 | 248.6419 | **K** | ***1378.5298*** | 689.7685 | 1361.5032 | 681.2552 | 1360.5192 | 680.7632 | **10** |
| **5** | **657.3454** | 329.1763 | 640.3188 | 320.6631 | 639.3348 | 320.1710 | **E** | ***1250.4348*** | 625.7210 |  |  | 1232.4242 | 616.7158 | **9** |
| **6** | 800.4036 | 400.7055 | 783.3771 | 392.1922 | 782.3931 | 391.7002 | **E** | ***1107.3766*** | 554.1919 |  |  | 1089.3660 | 545.1866 | **8** |
| **7** | 967.4020 | 484.2046 | 950.3754 | 475.6914 | 949.3914 | 475.1993 | **S** | ***964.3183*** | 482.6628 |  |  | 946.3078 | 473.6575 | **7** |
| **8** | **1096.4446** | 548.7259 | 1079.4180 | 540.2127 | 1078.4340 | 539.7206 | **D** | 797.3200 | 399.1636 |  |  | 779.3094 | 390.1583 | **6** |
| **9** | **1239.5028** | 620.2550 | 1222.4763 | 611.7418 | 1221.4923 | 611.2498 | **E** | ***668.2774*** | 334.6423 |  |  | 650.2668 | 325.6370 | **5** |
| **10** | **1382.5611** | 691.7842 | 1365.5345 | 683.2709 | 1364.5505 | 682.7789 | **E** | ***525.2191*** | 263.1132 |  |  | 507.2086 | 254.1079 | **4** |
| **11** | **1511.6037** | 756.3055 | 1494.5771 | 747.7922 | 1493.5931 | 747.3002 | **D** | ***382.1609*** | 191.5841 |  |  | 364.1503 | 182.5788 | **3** |
| **12** | 1674.6670 | 837.8371 | 1657.6404 | 829.3239 | 1656.6564 | 828.8318 | **Y** | ***253.1183*** | 127.0628 |  |  |  |  | **2** |
| **13** |  |  |  |  |  |  | **G** | 90.0550 | 45.5311 |  |  |  |  | **1** |

**ETD** MS/MS Fragmentation of **VEEKEESDEEDYG**
Found in **RLA02_ARATH**, 60S acidic ribosomal protein P0-2 OS=Arabidopsis thaliana GN=RPP0B PE=1 SV=2

Match to Query 1: 1762.525448 from(882.270000,2+)


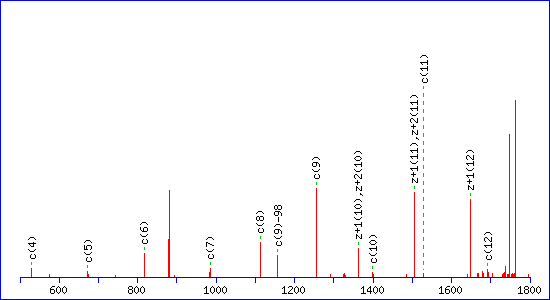


**Monoisotopic mass of neutral peptide Mr(calc):** 1762.7074

**Fixed modifications:** Methyl (C-term),Methyl (DE)

**Variable modifications:**

**S7 :** Phospho (ST), with neutral losses 0.0000(shown in table), 97.9769

**Ions Score:** 68 **Expect:** 8.1e-05

**Matches :** 15/144 fragment ions using 22 most intense peaks

| **#** | **c** | **c^++^** | **Seq.** | **y** | **y^++^** | **z+1** | **z+1^++^** | **z+2** | **z+2^++^** | **#** |
| --- | --- | --- | --- | --- | --- | --- | --- | --- | --- | --- |
| **1** | 117.1022 | 59.0548 | **V** |  |  |  |  |  |  | **13** |
| **2** | 260.1605 | 130.5839 | **E** | 1664.6463 | 832.8268 | **1648.6275** | 824.8174 | 1649.6354 | 825.3213 | **12** |
| **3** | 403.2187 | 202.1130 | **E** | 1521.5880 | 761.2976 | **1505.5693** | 753.2883 | 1506.5771 | 753.7922 | **11** |
| **4** | ***531.3137*** | 266.1605 | **K** | 1378.5298 | 689.7685 | **1362.5110** | 681.7592 | 1363.5189 | 682.2631 | **10** |
| **5** | ***674.3719*** | 337.6896 | **E** | 1250.4348 | 625.7210 | 1234.4161 | 617.7117 | 1235.4239 | 618.2156 | **9** |
| **6** | ***817.4302*** | 409.2187 | **E** | 1107.3766 | 554.1919 | 1091.3578 | 546.1826 | 1092.3657 | 546.6865 | **8** |
| **7** | ***984.4285*** | 492.7179 | **S** | 964.3183 | 482.6628 | 948.2996 | 474.6534 | 949.3074 | 475.1573 | **7** |
| **8** | ***1113.4711*** | 557.2392 | **D** | 797.3200 | 399.1636 | 781.3012 | 391.1543 | 782.3091 | 391.6582 | **6** |
| **9** | ***1256.5294*** | 628.7683 | **E** | 668.2774 | 334.6423 | 652.2586 | 326.6330 | 653.2665 | 327.1369 | **5** |
| **10** | ***1399.5876*** | 700.2974 | **E** | 525.2191 | 263.1132 | 509.2004 | 255.1038 | 510.2082 | 255.6077 | **4** |
| **11** | ***1528.6302*** | 764.8187 | **D** | 382.1609 | 191.5841 | 366.1422 | 183.5747 | 367.1500 | 184.0786 | **3** |
| **12** | ***1691.6935*** | 846.3504 | **Y** | 253.1183 | 127.0628 | 237.0996 | 119.0534 | 238.1074 | 119.5573 | **2** |
| **13** |  |  | **G** | 90.0550 | 45.5311 | 74.0362 | 37.5218 | 75.0441 | 38.0257 | **1** |

**CID** MS/MS Fragmentation of **ALSTSKPDPVVEDQA**
Found in **RS23_ARATH**, 40S ribosomal protein S2-3 OS=Arabidopsis thaliana GN=RPS2C PE=1 SV=2

Match to Query 1: 1691.665448 from(846.840000,2+)


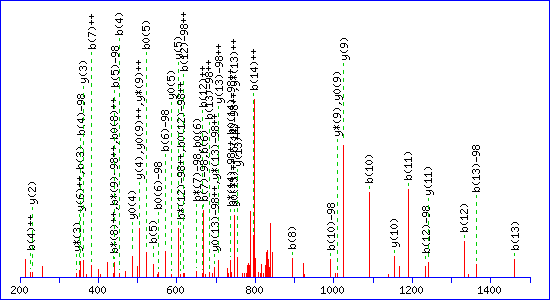


**Monoisotopic mass of neutral peptide Mr(calc):** 1691.8019

**Fixed modifications:** Methyl (C-term),Methyl (DE)

**Variable modifications:**

**S3 :** Phospho (ST), with neutral losses 0.0000(shown in table), 97.9769

**Ions Score:** 48 **Expect:** 0.00018

**Matches :** 56/226 fragment ions using 70 most intense peaks

| **#** | **b** | **b++** | **b*** | **b*++** | **b0** | **b0++** | **Seq.** | **y** | **y++** | **y*** | **y*++** | **y0** | **y0++** | **#** |
| --- | --- | --- | --- | --- | --- | --- | --- | --- | --- | --- | --- | --- | --- | --- |
| **1** | 72.0444 | 36.5258 |  |  |  |  | **A** |  |  |  |  |  |  | **15** |
| **2** | 185.1285 | 93.0679 |  |  |  |  | **L** | 1621.7721 | 811.3897 | 1604.7455 | 802.8764 | 1603.7615 | 802.3844 | **14** |
| **3** | ***352.1268*** | 176.5670 |  |  | 334.1162 | 167.5618 | **S** | 1508.6880 | 754.8476 | 1491.6614 | 746.3344 | 1490.6774 | 745.8424 | **13** |
| **4** | ***453.1745*** | **227.0909** |  |  | 435.1639 | 218.0856 | **T** | 1341.6896 | 671.3485 | 1324.6631 | 662.8352 | 1323.6791 | 662.3432 | **12** |
| **5** | ***540.2065*** | 270.6069 |  |  | 522.1960 | 261.6016 | **S** | **1240.6420** | 620.8246 | 1223.6154 | 612.3113 | 1222.6314 | 611.8193 | **11** |
| **6** | ***668.3015*** | 334.6544 | 651.2749 | 326.1411 | 650.2909 | 325.6491 | **K** | **1153.6099** | 577.3086 | 1136.5834 | 568.7953 | 1135.5994 | 568.3033 | **10** |
| **7** | 765.3542 | **383.1808** | 748.3277 | 374.6675 | 747.3437 | 374.1755 | **P** | **1025.5150** | 513.2611 | 1008.4884 | 504.7478 | 1007.5044 | 504.2558 | **9** |
| **8** | ***894.3968*** | 447.7021 | 877.3703 | 439.1888 | 876.3863 | 438.6968 | **D** | 928.4622 | 464.7347 | 911.4357 | 456.2215 | 910.4516 | 455.7295 | **8** |
| **9** | 991.4496 | 496.2284 | 974.4231 | 487.7152 | 973.4390 | 487.2232 | **P** | 799.4196 | 400.2134 | 782.3931 | 391.7002 | 781.4090 | 391.2082 | **7** |
| **10** | ***1090.5180*** | 545.7626 | 1073.4915 | 537.2494 | 1072.5075 | 536.7574 | **V** | 702.3668 | 351.6871 | 685.3403 | 343.1738 | 684.3563 | 342.6818 | **6** |
| **11** | ***1189.5864*** | 595.2969 | 1172.5599 | 586.7836 | 1171.5759 | 586.2916 | **V** | **603.2984** | 302.1529 | 586.2719 | 293.6396 | 585.2879 | 293.1476 | **5** |
| **12** | ***1332.6447*** | **666.8260** | 1315.6181 | 658.3127 | 1314.6341 | 657.8207 | **E** | **504.2300** | 252.6186 | 487.2035 | 244.1054 | 486.2195 | 243.6134 | **4** |
| **13** | ***1461.6873*** | 731.3473 | 1444.6607 | 722.8340 | 1443.6767 | 722.3420 | **D** | **361.1718** | 181.0895 | 344.1452 | 172.5763 | 343.1612 | 172.0842 | **3** |
| **14** | 1589.7458 | **795.3766** | 1572.7193 | 786.8633 | 1571.7353 | 786.3713 | **Q** | **232.1292** | 116.5682 | 215.1026 | 108.0550 |  |  | **2** |
| **15** |  |  |  |  |  |  | **A** | 104.0706 | 52.5389 |  |  |  |  | **1** |

**ETD** MS/MS Fragmentation of **ALSTSKPDPVVEDQA**
Found in **RS23_ARATH**, 40S ribosomal protein S2-3 OS=Arabidopsis thaliana GN=RPS2C PE=1 SV=2

Match to Query 1: 1691.665448 from(846.840000,2+)


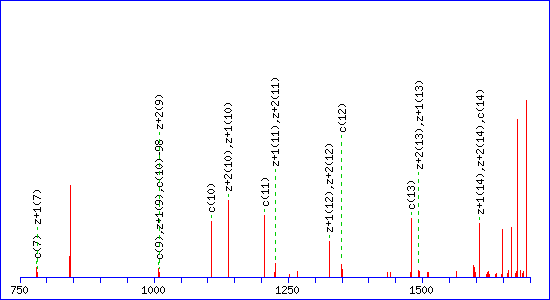


**Monoisotopic mass of neutral peptide Mr(calc):** 1691.8019

**Fixed modifications:** Methyl (C-term),Methyl (DE)

**Variable modifications:**

**S3 :** Phospho (ST), with neutral losses 0.0000(shown in table), 97.9769

**Ions Score:** 61 **Expect:** 1.2e-05

**Matches :** 21/148 fragment ions using 31 most intense peaks

| **#** | **c** | **c++** | **Seq.** | **y** | **y++** | **z+1** | **z+1++** | **z+2** | **z+2++** | **#** |
| --- | --- | --- | --- | --- | --- | --- | --- | --- | --- | --- |
| **1** | 89.0709 | 45.0391 | **A** |  |  |  |  |  |  | **15** |
| **2** | 202.1550 | 101.5811 | **L** | 1621.7721 | 811.3897 | ***1605.7533*** | 803.3803 | **1606.7612** | 803.8842 | **14** |
| **3** | 369.1534 | 185.0803 | **S** | 1508.6880 | 754.8476 | ***1492.6693*** | 746.8383 | **1493.6771** | 747.3422 | **13** |
| **4** | 470.2010 | 235.6042 | **T** | 1341.6896 | 671.3485 | ***1325.6709*** | 663.3391 | **1326.6787** | 663.8430 | **12** |
| **5** | 557.2331 | 279.1202 | **S** | 1240.6420 | 620.8246 | ***1224.6232*** | 612.8153 | **1225.6311** | 613.3192 | **11** |
| **6** | 685.3280 | 343.1677 | **K** | 1153.6099 | 577.3086 | ***1137.5912*** | 569.2992 | **1138.5990** | 569.8032 | **10** |
| **7** | ***782.3808*** | 391.6940 | **P** | 1025.5150 | 513.2611 | ***1009.4962*** | 505.2518 | **1010.5041** | 505.7557 | **9** |
| **8** | 911.4234 | 456.2153 | **D** | 928.4622 | 464.7347 | 912.4435 | 456.7254 | 913.4513 | 457.2293 | **8** |
| **9** | ***1008.4762*** | 504.7417 | **P** | 799.4196 | 400.2134 | ***783.4009*** | 392.2041 | 784.4087 | 392.7080 | **7** |
| **10** | ***1107.5446*** | 554.2759 | **V** | 702.3668 | 351.6871 | 686.3481 | 343.6777 | 687.3559 | 344.1816 | **6** |
| **11** | ***1206.6130*** | 603.8101 | **V** | 603.2984 | 302.1529 | 587.2797 | 294.1435 | 588.2875 | 294.6474 | **5** |
| **12** | ***1349.6712*** | 675.3392 | **E** | 504.2300 | 252.6186 | 488.2113 | 244.6093 | 489.2191 | 245.1132 | **4** |
| **13** | ***1478.7138*** | 739.8605 | **D** | 361.1718 | 181.0895 | 345.1531 | 173.0802 | 346.1609 | 173.5841 | **3** |
| **14** | ***1606.7724*** | 803.8898 | **Q** | 232.1292 | 116.5682 | 216.1105 | 108.5589 | 217.1183 | 109.0628 | **2** |
| **15** |  |  | **A** | 104.0706 | 52.5389 | 88.0519 | 44.5296 | 89.0597 | 45.0335 | **1** |

**CID** MS/MS Fragmentation of **AGENASAEE**
Found in **RL291_ARATH**, 60S ribosomal protein L29-1 OS=Arabidopsis thaliana GN=RPL29A PE=1 SV=1

Match to Query 4: 1012.265448 from(507.140000,2+)


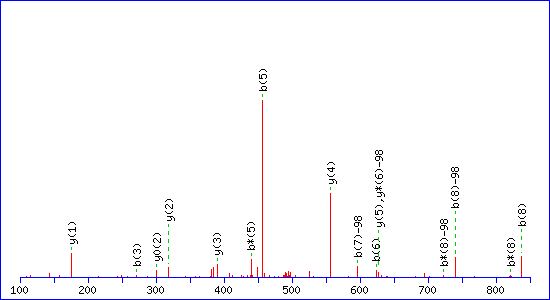


**Monoisotopic mass of neutral peptide Mr(calc):** 1012.3750

**Fixed modifications:** Methyl (C-term),Methyl (DE)

**Variable modifications:**

**S6 :** Phospho (ST), with neutral losses 0.0000(shown in table), 97.9769

**Ions Score:** 47 **Expect:** 0.00022

**Matches :** 16/120 fragment ions using 16 most intense peaks

| **#** | **b** | **b^++^** | **b*** | **b*^++^** | **b^0^** | **b^0++^** | **Seq.** | **y** | **y^++^** | **y*** | **y*^++^** | **y^0^** | **y^0++^** | **#** |
| --- | --- | --- | --- | --- | --- | --- | --- | --- | --- | --- | --- | --- | --- | --- |
| **1** | 72.0444 | 36.5258 |  |  |  |  | **A** |  |  |  |  |  |  | **9** |
| **2** | 129.0659 | 65.0366 |  |  |  |  | **G** | 942.3452 | 471.6762 | 925.3186 | 463.1630 | 924.3346 | 462.6710 | **8** |
| **3** | ***272.1241*** | 136.5657 |  |  | 254.1135 | 127.5604 | **E** | 885.3237 | 443.1655 | 868.2972 | 434.6522 | 867.3132 | 434.1602 | **7** |
| **4** | 386.1670 | 193.5872 | 369.1405 | 185.0739 | 368.1565 | 184.5819 | **N** | 742.2655 | 371.6364 | 725.2389 | 363.1231 | 724.2549 | 362.6311 | **6** |
| **5** | ***457.2041*** | 229.1057 | 440.1776 | 220.5924 | 439.1936 | 220.1004 | **A** | ***628.2226*** | 314.6149 |  |  | 610.2120 | 305.6096 | **5** |
| **6** | ***624.2025*** | 312.6049 | 607.1759 | 304.0916 | 606.1919 | 303.5996 | **S** | ***557.1854*** | 279.0964 |  |  | 539.1749 | 270.0911 | **4** |
| **7** | 695.2396 | 348.1234 | 678.2131 | 339.6102 | 677.2290 | 339.1182 | **A** | ***390.1871*** | 195.5972 |  |  | 372.1765 | 186.5919 | **3** |
| **8** | ***838.2979*** | 419.6526 | 821.2713 | 411.1393 | 820.2873 | 410.6473 | **E** | ***319.1500*** | 160.0786 |  |  | 301.1394 | 151.0733 | **2** |
| **9** |  |  |  |  |  |  | **E** | ***176.0917*** | 88.5495 |  |  | 158.0812 | 79.5442 | **1** |

**ETD** MS/MS Fragmentation of **AGENASAEE**
Found in **RL291_ARATH**, 60S ribosomal protein L29-1 OS=Arabidopsis thaliana GN=RPL29A PE=1 SV=1

Match to Query 5: 1012.285448 from(507.150000,2+)


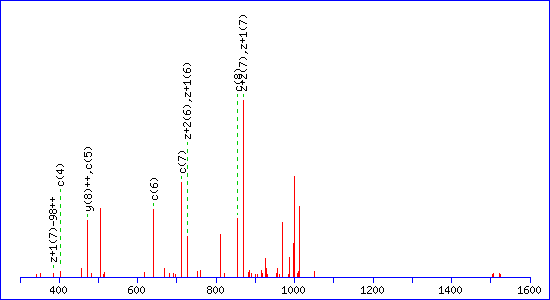


**Monoisotopic mass of neutral peptide Mr(calc):** 1012.3750

**Fixed modifications:** Methyl (C-term),Methyl (DE)

**Variable modifications:**

**S6 :** Phospho (ST), with neutral losses 0.0000(shown in table), 97.9769

**Ions Score:** 35 **Expect:** 0.0039

**Matches :** 11/100 fragment ions using 14 most intense peaks

| **#** | **c** | **c^++^** | **Seq.** | **y** | **y^++^** | **z+1** | **z+1^++^** | **z+2** | **z+2^++^** | **#** |
| --- | --- | --- | --- | --- | --- | --- | --- | --- | --- | --- |
| **1** | 89.0709 | 45.0391 | **A** |  |  |  |  |  |  | **9** |
| **2** | 146.0924 | 73.5498 | **G** | 942.3452 | 471.6762 | 926.3265 | 463.6669 | 927.3343 | 464.1708 | **8** |
| **3** | 289.1506 | 145.0790 | **E** | 885.3237 | 443.1655 | **869.3050** | 435.1561 | **870.3128** | 435.6601 | **7** |
| **4** | ***403.1936*** | 202.1004 | **N** | 742.2655 | 371.6364 | **726.2468** | 363.6270 | **727.2546** | 364.1309 | **6** |
| **5** | ***474.2307*** | 237.6190 | **A** | 628.2226 | 314.6149 | 612.2038 | 306.6056 | 613.2117 | 307.1095 | **5** |
| **6** | ***641.2290*** | 321.1182 | **S** | 557.1854 | 279.0964 | 541.1667 | 271.0870 | 542.1746 | 271.5909 | **4** |
| **7** | ***712.2662*** | 356.6367 | **A** | 390.1871 | 195.5972 | 374.1684 | 187.5878 | 375.1762 | 188.0917 | **3** |
| **8** | ***855.3244*** | 428.1658 | **E** | 319.1500 | 160.0786 | 303.1313 | 152.0693 | 304.1391 | 152.5732 | **2** |
| **9** |  |  | **E** | 176.0917 | 88.5495 | 160.0730 | 80.5401 | 161.0808 | 81.0441 | **1** |

**CID** MS/MS Fragmentation of **AGENASAEE**
Found in **RL291_ARATH**, 60S ribosomal protein L29-1 OS=Arabidopsis thaliana GN=RPL29A PE=1 SV=1

Match to Query 1: 1012.242724 from(1013.250000,1+)


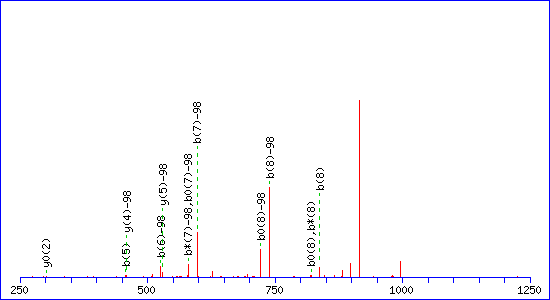


**Monoisotopic mass of neutral peptide Mr(calc):** 1012.3750

**Fixed modifications:** Methyl (C-term),Methyl (DE)

**Variable modifications:**

**S6 :** Phospho (ST), with neutral losses 97.9769(shown in table), 0.0000

**Ions Score:** 27 **Expect:** 0.016

**Matches :** 13/60 fragment ions using 16 most intense peaks

| **#** | **b** | **b*** | **b^0^** | **Seq.** | **y** | **y*** | **y^0^** | **#** |
| --- | --- | --- | --- | --- | --- | --- | --- | --- |
| **1** | 72.0444 |  |  | **A** |  |  |  | **9** |
| **2** | 129.0659 |  |  | **G** | 844.3683 | 827.3418 | 826.3577 | **8** |
| **3** | 272.1241 |  | 254.1135 | **E** | 787.3468 | 770.3203 | 769.3363 | **7** |
| **4** | 386.1670 | 369.1405 | 368.1565 | **N** | 644.2886 | 627.2620 | 626.2780 | **6** |
| **5** | ***457.2041*** | 440.1776 | 439.1936 | **A** | **530.2457** |  | 512.2351 | **5** |
| **6** | ***526.2256*** | 509.1991 | 508.2150 | **S** | **459.2086** |  | 441.1980 | **4** |
| **7** | ***597.2627*** | 580.2362 | 579.2521 | **A** | 390.1871 |  | 372.1765 | **3** |
| **8** | ***740.3210*** | 723.2944 | 722.3104 | **E** | 319.1500 |  | 301.1394 | **2** |
| **9** |  |  |  | **E** | 176.0917 |  | 158.0812 | **1** |
