# Supplementary material for: Differential Phosphorylation of Ribosomal Proteins in Arabidopsis thaliana Plants during Day and Night
Source: PLoS One. 2011 Dec 16;6(12):e29307. doi: 10.1371/journal.pone.0029307 (PMC3241707; doi:10.1371/journal.pone.0029307)
Supplement: Table S1 — Ribosomal proteins identified by nano-LC-MS/MS. A list of cytosolic ribosomal proteins obtained using Scaffold proteomic program from combined MASCOT identifications in all ribosomal protein preparations. Gene locus, number of sequenced peptides and number of unique sequenced peptides are indicated. (DOC) [file pone.0029307.s002.doc]

| **Table S1. Ribosomal proteins identified by nano-LC-MS/MS** | | | |
| --- | --- | --- | --- |
| Identified Proteins | Gene locus | Peptides  sequenced | Peptides  unique |
| 40S ribosomal protein Sa-1 | At1g72370 | 9 | 6 |
| 40S ribosomal protein S02-1 | At1g58380 | 5 | 0 |
| 40S ribosomal protein S02-3 | At2g41840 | 6 | 3 |
| 40S ribosomal protein S03-1 | At2g31610 | 11 | 2 |
| 40S ribosomal protein S03-2 | At3g53870 | 10 | 2 |
| 40S ribosomal protein S03-3 | At5g35530 | 9 | 2 |
| 40S ribosomal protein S03a-1 | At3g04840 | 6 | 4 |
| 40S ribosomal protein S03a-2 | At4g34670 | 7 | 5 |
| 40S ribosomal protein S04-2 | At5g07090 | 10 | 0 |
| 40S ribosomal protein S05-2 | At3g11940 | 4 | 2 |
| 40S ribosomal protein S06-1 | At4g31700 | 6 | 2 |
| 40S ribosomal protein S06-2 | At5g10360 | 6 | 3 |
| 40S ribosomal protein S07-1 | At1g48830 | 4 | 4 |
| 40S ribosomal protein S07-2 | At3g02560 | 5 | 4 |
| 40S ribosomal protein S07-3 | At5g16130 | 4 | 3 |
| 40S ribosomal protein S08-1 | At5g20290 | 7 | 6 |
| 40S ribosomal protein S09-1 | At5g15200 | 9 | 4 |
| 40S ribosomal protein S10-2 | At5g41520 | 3 | 3 |
| 40S ribosomal protein S10-3 | At5g52650 | 3 | 0 |
| 40S ribosomal protein S11-3 | At5g23740 | 3 | 1 |
| 40S ribosomal protein S12-1 | At1g15930 | 3 | 2 |
| 40S ribosomal protein S12-2 | At2g32060 | 2 | 1 |
| 40S ribosomal protein S13-2 | At4g00100 | 5 | 1 |
| 40S ribosomal protein S14-2 | At3g11510 | 3 | 1 |
| 40S ribosomal protein S15-1 | At1g04270 | 3 | 0 |
| 40S ribosomal protein S15a-1 | At1g07770 | 5 | 1 |
| 40S ribosomal protein S16-3 | At5g18380 | 5 | 0 |
| 40S ribosomal protein S17-2 | At2g05220 | 4 | 1 |
| 40S ribosomal protein S18 | At1g22780 | 5 | 5 |
| 40S ribosomal protein S19-1 | At3g02080 | 3 | 1 |
| 40S ribosomal protein S20-1 | At3g45030 | 4 | 2 |
| 40S ribosomal protein S23-2 | At5g02960 | 2 | 0 |
| 40S ribosomal protein S24-1 | At3g04920 | 3 | 2 |
| 40S ribosomal protein S24-2 | At5g28060 | 3 | 2 |
| 40S ribosomal protein S25-3 | At4g34555 | 2 | 0 |
| 40S ribosomal protein S26-1 | At2g40590 | 2 | 0 |
| 40S ribosomal protein S27-1 | At2g45710 | 2 | 0 |
| 40S ribosomal protein S27a-2 | At2g47110 | 3 | 1 |
| 40S ribosomal protein S28-1 | At3g10090 | 1 | 1 |
| 40S ribosomal protein S28-2 | At5g64140 | 1 | 1 |
| 60S ribosomal protein L03-1 | At1g43170 | 12 | 10 |
| 60S ribosomal protein L04-1 | At3g09630 | 10 | 4 |
| 60S ribosomal protein L04-2 | At5g02870 | 7 | 2 |
| 60S ribosomal protein L05-1 | At3g25520 | 7 | 1 |
| 60S ribosomal protein L06-1 | At1g18540 | 7 | 3 |
| 60S ribosomal protein L06-2 | At1g74060 | 9 | 0 |
| 60S ribosomal protein L07-2 | At2g01250 | 10 | 5 |
| 60S ribosomal protein L07-3 | At2g44120 | 9 | 1 |
| 60S ribosomal protein L07a-1 | At2g47610 | 7 | 1 |
| 60S ribosomal protein L07a-2 | At3g62870 | 8 | 2 |
| 60S ribosomal protein L08-1 | At2g18020 | 5 | 2 |
| 60S ribosomal protein L09-1 | At1g33120 | 10 | 7 |
| 60S ribosomal protein L10-1 | At1g14320 | 5 | 1 |
| 60S ribosomal protein L10a-1 | At1g08360 | 3 | 0 |
| 60S ribosomal protein L10a-2 | At2g27530 | 4 | 2 |
| 60S ribosomal protein L11-2 | At3g58700 | 4 | 0 |
| 60S ribosomal protein L12-1 | At2g37190 | 5 | 1 |
| 60S ribosomal protein L13-1 | At3g49010 | 5 | 3 |
| 60S ribosomal protein L13-3 | At5g23900 | 4 | 2 |
| 60S ribosomal protein L13a-2 | At3g24830 | 3 | 1 |
| 60S ribosomal protein L14-2 | At4g27090 | 5 | 2 |
| 60S ribosomal protein L15-1 | At4g16720 | 5 | 0 |
| 60S ribosomal protein L17-1 | At1g27400 | 3 | 0 |
| 60S ribosomal protein L18-3 | At5g27850 | 6 | 2 |
| 60S ribosomal protein L18a-2 | At2g34480 | 7 | 2 |
| 60S ribosomal protein L19-1 | At1g02780 | 2 | 2 |
| 60S ribosomal protein L21-1 | At1g09590 | 2 | 0 |
| 60S ribosomal protein L22-2 | At3g05560 | 4 | 1 |
| 60S ribosomal protein L22-3 | At5g27770 | 5 | 2 |
| 60S ribosomal protein L23 | At2g33370 | 5 | 5 |
| 60S ribosomal protein L23a-1 | At2g39460 | 3 | 0 |
| 60S ribosomal protein L24-2 | At3g53020 | 3 | 1 |
| 60S ribosomal protein L26-1 | At3g49910 | 2 | 2 |
| 60S ribosomal protein L27-3 | At4g15000 | 4 | 0 |
| 60S ribosomal protein L27a-3 | At1g70600 | 4 | 0 |
| 60S ribosomal protein L28-1 | At2g19730 | 3 | 2 |
| Putative 60S ribosomal protein L30-1 | At1g36240 | 3 | 0 |
| 60S ribosomal protein L31-3 | At5g56710 | 2 | 0 |
| 60S ribosomal protein L32-1 | At4g18100 | 5 | 3 |
| 60S ribosomal protein L32-2 | At5g46430 | 4 | 2 |
| 60S ribosomal protein L34-2 | At1g69620 | 3 | 1 |
| 60S ribosomal protein L35-1 | At3g09500 | 2 | 1 |
| 60S ribosomal protein L35a-4 | At3g55750 | 2 | 0 |
| 60S ribosomal protein L36-2 | At3g53740 | 1 | 1 |
| 60S ribosomal protein L37-3 | At3g16080 | 2 | 0 |
| Putative 60S ribosomal protein L37a-1 | At3g10950 | 2 | 0 |
| 60S ribosomal protein L38 | At2g43460 | 4 | 4 |
| 60S acidic ribosomal protein P0-2 | At3g09200 | 7 | 4 |
| 60S acidic ribosomal protein P1-1 | At1g01100 | 5 | 2 |
| 60S acidic ribosomal protein P1-2 | At4g00810 | 5 | 3 |
| 60S acidic ribosomal protein P1-3 | At5g47700 | 5 | 2 |
| 60S acidic ribosomal protein P2-1 | At2g27720 | 4 | 2 |
| 60S acidic ribosomal protein P2-2 | At2g27710 | 5 | 3 |
| 60S acidic ribosomal protein P3-2 | At5g57290 | 3 | 2 |
| RACK1A | At1g18080 | 6 | 6 |
