# Supplementary material for: Differential Phosphorylation of Ribosomal Proteins in Arabidopsis thaliana Plants during Day and Night
Source: PLoS One. 2011 Dec 16;6(12):e29307. doi: 10.1371/journal.pone.0029307 (PMC3241707; doi:10.1371/journal.pone.0029307)
Supplement: Table S2 — Spectral counts for ribosomal proteins in four biological samples (two day and two night) calculated using Scaffold proteomic program. (DOC) [file pone.0029307.s003.doc]

| **Table S2. Spectral counts for ribosomal proteins in four biological samples (two day and two night) calculated using Scaffold proteomic** **program** | | | | |
| --- | --- | --- | --- | --- |
| Identified Proteins | Spectral counts | | | |
|  | Day 1 | Day 2 | Night 1 | Night 2 |
| 40S ribosomal protein Sa-1 | 19.58 | 13.19 | 11.20 | 18.17 |
| 40S ribosomal protein S02-1 | 13.29 | 16.15 | 10.53 | 9.98 |
| 40S ribosomal protein S02-3 | 16.99 | 12.49 | 16.48 | 14.35 |
| 40S ribosomal protein S03-1 | 26.15 | 28.89 | 20.93 | 20.85 |
| 40S ribosomal protein S03-2 | 23.79 | 4.67 | 17.95 | 15.29 |
| 40S ribosomal protein S03-3 | 19.18 | 26.35 | 13.96 | 1.00 |
| 40S ribosomal protein S03a-1 | 4.67 | 13.95 | 6.70 | 9.98 |
| 40S ribosomal protein S03a-2 | 5.45 | 12.75 | 4.79 | 7.98 |
| 40S ribosomal protein S04-2 | 26.99 | 19.57 | 21.03 | 17.95 |
| 40S ribosomal protein S05-2 | 26.93 | 18.28 | 29.03 | 23.67 |
| 40S ribosomal protein S06-1 | 16.55 | 11.90 | 11.96 | 16.26 |
| 40S ribosomal protein S06-2 | 14.81 | 11.05 | 15.3 | 13.37 |
| 40S ribosomal protein S07-1 | 6.95 | 6.80 | 5.98 | 6.11 |
| 40S ribosomal protein S07-2 | 7.06 | 4.67 | 1.99 | 4.17 |
| 40S ribosomal protein S07-3 | 5.59 | 4.08 | 5.74 | 4.16 |
| 40S ribosomal protein S08-1 | 11.42 | 15.33 | 11.48 | 11.25 |
| 40S ribosomal protein S09-1 | 21.78 | 21.23 | 17.94 | 19.83 |
| 40S ribosomal protein S10-2 | 6.11 | 2.54 | 5.74 | 1.99 |
| 40S ribosomal protein S10-3 | 8.72 | 6.24 | 6.98 | 6.26 |
| 40S ribosomal protein S11-3 | 5.23 | 3.23 | 2.99 | 2.99 |
| 40S ribosomal protein S12-1 | 6.97 | 5.96 | 6.98 | 5.74 |
| 40S ribosomal protein S12-2 | 5.23 | 6.80 | 6.98 | 6.69 |
| 40S ribosomal protein S13-2 | 10.46 | 10.20 | 7.65 | 9.98 |
| 40S ribosomal protein S14-2 | 11.42 | 8.73 | 10.97 | 8.24 |
| 40S ribosomal protein S15-1 | 9.05 | 13.10 | 9.20 | 7.08 |
| 40S ribosomal protein S15a-1 | 9.34 | 4.87 | 9.38 | 4.98 |
| 40S ribosomal protein S16-3 | 14.81 | 22.58 | 16.48 | 19.5 |
| 40S ribosomal protein S17-2 | 7.65 | 4.08 | 4.17 | 3.83 |
| 40S ribosomal protein S18 | 23.94 | 27.72 | 19.67 | 25.68 |
| 40S ribosomal protein S19-1 | 10.46 | 8.63 | 10.96 | 11.36 |
| 40S ribosomal protein S20-1 | 5.24 | 7.83 | 4.78 | 4.99 |
| 40S ribosomal protein S23-2 | 3.23 | 4.24 | 2.99 | 2.09 |
| 40S ribosomal protein S24-1 | 7.83 | 7.64 | 5.98 | 7.65 |
| 40S ribosomal protein S24-2 | 4.35 | 5.94 | 4.98 | 6.69 |
| 40S ribosomal protein S25-3 | 2.60 | 2.44 | 1.99 | 3.99 |
| 40S ribosomal protein S26-1 | 3.49 | 4.23 | 3.99 | 5.74 |
| 40S ribosomal protein S27-1 | 6.24 | 5.73 | 2.84 | 3.06 |
| 40S ribosomal protein S27a-2 | 3.23 | 5.10 | 4.78 | 3.12 |
| 40S ribosomal protein S28-2 | 2.60 | 2.54 | 2.99 | 1.91 |
| 60S ribosomal protein L03-1 | 19.38 | 17.87 | 17.49 | 18.49 |
| 60S ribosomal protein L04-1 | 17.88 | 21.22 | 25.84 | 16.43 |
| 60S ribosomal protein L04-2 | 23.04 | 25.08 | 23.82 | 17.49 |
| 60S ribosomal protein L05-1 | 20.90 | 17.73 | 20.93 | 18.17 |
| 60S ribosomal protein L06-1 | 16.58 | 14.45 | 17.95 | 17.76 |
| 60S ribosomal protein L06-2 | 18.3 | 16.15 | 21.93 | 17.74 |
| 60S ribosomal protein L07-2 | 29.93 | 28.82 | 28.92 | 32.92 |
| 60S ribosomal protein L07-3 | 20.67 | 19.09 | 20.63 | 24.67 |
| 60S ribosomal protein L07a-1 | 12.21 | 17.84 | 13.95 | 10.97 |
| 60S ribosomal protein L07a-2 | 13.09 | 8.26 | 13.95 | 9.57 |
| 60S ribosomal protein L08-1 | 12.42 | 8.63 | 7.23 | 10.30 |
| 60S ribosomal protein L09-1 | 27.01 | 28.89 | 25.92 | 32.52 |
| 60S ribosomal protein L10-1 | 14.83 | 14.42 | 13.96 | 14.61 |
| 60S ribosomal protein L10a-1 | 9.60 | 5.17 | 8.97 | 5.16 |
| 60S ribosomal protein L10a-2 | 11.35 | 13.58 | 12.96 | 10.43 |
| 60S ribosomal protein L11-2 | 14.81 | 13.60 | 13.39 | 12.52 |
| 60S ribosomal protein L12-1 | 13.60 | 25.83 | 14.59 | 26.95 |
| 60S ribosomal protein L13-1 | 18.87 | 13.95 | 15.52 | 10.96 |
| 60S ribosomal protein L13-3 | 9.35 | 1.00 | 5.74 | 4.98 |
| 60S ribosomal protein L13a-2 | 6.11 | 4.17 | 8.97 | 4.09 |
| 60S ribosomal protein L14-2 | 12.72 | 13.51 | 13.54 | 5.16 |
| 60S ribosomal protein L15-1 | 9.34 | 10.46 | 7.28 | 9.30 |
| 60S ribosomal protein L17-1 | 7.83 | 5.94 | 8.97 | 7.30 |
| 60S ribosomal protein L18-3 | 14.81 | 21.66 | 16.95 | 23.82 |
| 60S ribosomal protein L18a-2 | 13.92 | 16.98 | 13.95 | 15.64 |
| 60S ribosomal protein L19-1 | 3.18 | 4.87 | 17.6 | 13.37 |
| 60S ribosomal protein L21-1 | 5.23 | 5.10 | 5.98 | 4.17 |
| 60S ribosomal protein L22-2 | 12.21 | 8.50 | 10.43 | 9.97 |
| 60S ribosomal protein L22-3 | 10.46 | 8.50 | 8.97 | 8.35 |
| 60S ribosomal protein L23 | 12.21 | 11.05 | 13.39 | 6.98 |
| 60S ribosomal protein L23a-1 | 4.37 | 4.88 | 3.99 | 4.99 |
| 60S ribosomal protein L24-2 | 8.72 | 8.43 | 6.98 | 8.24 |
| 60S ribosomal protein L26-1 | 3.49 | 3.08 | 2.86 | 2.07 |
| 60S ribosomal protein L27-3 | 11.32 | 11.9 | 7.97 | 9.38 |
| 60S ribosomal protein L27a-3 | 13.95 | 9.75 | 8.6 | 11.38 |
| 60S ribosomal protein L28-1 | 8.72 | 7.51 | 6.98 | 9.19 |
| Putative 60S ribosomal protein L30-1 | 10.44 | 9.34 | 8.97 | 10.42 |
| 60S ribosomal protein L31-3 | 2.63 | 2.09 | 4.19 | 1.00 |
| 60S ribosomal protein L32-1 | 7.25 | 2.09 | 8.62 | 2.07 |
| 60S ribosomal protein L32-2 | 8.74 | 6.80 | 5.98 | 5.22 |
| 60S ribosomal protein L34-2 | 3.40 | 5.69 | 4.16 | 5.13 |
| 60S ribosomal protein L35-1 | 3.23 | 1.70 | 2.28 | 4.17 |
| 60S ribosomal protein L35a-4 | 2.60 | 3.38 | 1.99 | 3.12 |
| 60S ribosomal protein L36-2 | 2.56 | 2.22 | 2.09 | 2.86 |
| 60S ribosomal protein L37-3 | 2.44 | 2.09 | 1.99 | 2.07 |
| Putative 60S ribosomal protein L37a-1 | 3.38 | 3.23 | 2.09 | 3.81 |
| 60S ribosomal protein L38 | 10.49 | 8.05 | 9.97 | 9.14 |
| 60S acidic ribosomal protein P0-2 | 14.81 | 22.92 | 25.91 | 27.08 |
| 60S acidic ribosomal protein P1-1 | 13.92 | 15.31 | 17.95 | 12.33 |
| 60S acidic ribosomal protein P2-1 | 17.44 | 19.77 | 15.43 | 16.48 |
| 60S acidic ribosomal protein P2-2 | 15.69 | 29.01 | 17.94 | 15.47 |
| 60S acidic ribosomal protein P3-2 | 9.58 | 8.50 | 10.96 | 5.21 |
| RACK1A | 6.80 | 2.44 | 4.17 | 4.99 |
